# Deciphering African late middle Pleistocene hominin diversity and the origin of our species

**DOI:** 10.1038/s41467-019-11213-w

**Published:** 2019-09-10

**Authors:** Aurélien Mounier, Marta Mirazón Lahr

**Affiliations:** 1 UMR 7194, CNRS, Muséum national d’Histoire naturelle, Musée de l’Homme, 17 place du Trocadéro et du 11 novembre, 75016 Paris, France; 2 Leverhulme Centre for Human Evolutionary Studies, Department of Archaeology, University of Cambridge, Fitzwilliam Street, Cambridge, CB2 1QH United Kingdom; 3 Turkana Basin Institute, Hardy Post, Ushirika Road, Karen, P.O. Box 24467, Nairobi, 00502 Kenya

**Keywords:** Palaeontology, Biological anthropology, Phylogenetics

## Abstract

The origin of *Homo sapiens* remains a matter of debate. The extent and geographic patterning of morphological diversity among Late Middle Pleistocene (LMP) African hominins is largely unknown, thus precluding the definition of boundaries of variability in early *H. sapiens* and the interpretation of individual fossils. Here we use a phylogenetic modelling method to predict possible morphologies of a last common ancestor of all modern humans, which we compare to LMP African fossils (KNM-ES 11693, Florisbad, Irhoud 1, Omo II, and LH18). Our results support a complex process for the evolution of *H. sapiens*, with the recognition of different, geographically localised, populations and lineages in Africa – not all of which contributed to our species’ origin. Based on the available fossils, *H. sapiens* appears to have originated from the coalescence of South and, possibly, East-African source populations, while North-African fossils may represent a population which introgressed into Neandertals during the LMP.

## Abstract (web summary)

Late Middle Pleistocene (LMP) hominin fossils are scarce, limiting reconstruction of human evolution during this key period. Here, the authors use phylogenetic modelling to predict the modern human last common ancestor’s morphology and inform hypotheses of human origins by comparison to LMP fossils.

## Introduction

The history and evolution of the genus *Homo* has been the subject of continuous debates^1,2^. Recent fossil discoveries^3,4^, new analytical techniques^5,6^ and major developments in ancient genomics^7,8^ have considerably enhanced our understanding of the evolution of our genus, modifying profoundly the palaeoanthropological theoretical framework. Therefore, models for the origin of our species, *H. sapiens*, have moved away from the confrontation of two extreme antagonistic points of view: (1) the Multiregional Model of modern human origins implying the gradual evolution of global archaic hominin populations towards a modern human morphology over the course of the last 2 million years;^9,10^ and (2) the hypothesis of a unique Out of Africa event^1^, also known as the Recent African Origin (RAO) model, implying a single African origin (200,000–100,000 years ago (ka)) of modern humans and subsequent replacement of all archaic populations. Subsequent models to address the complex evolutionary geography of late Quaternary hominins were proposed, including multiple dispersals out of Africa and the role of population structure in Africa in the processes of diversification^11^. Along with the understanding of the evolutionary mechanisms that gave rise to the Neandertals in Europe^12^, and a growing number of genetic studies^13,14^, those models paved the way for a shift of emphasis from replacement to the potentially varied demographic and genetic outcomes of recent hominin interactions^7,9,15–17^.

Nevertheless, many unanswered questions remain, partly because of the scarcity of Late Middle Pleistocene (LMP, from 350 to 130 ka) African fossils which is a major constraint to any study of the African LMP fossil record. In Northern Africa, the site of Jebel Irhoud has yielded multiple fossils since the 1960s^18^, including a complete skull (Irhoud 1), originally dated to 130–190 ka^19^. Recent excavations at the site have yielded additional fossils (in particular a partial upper face, Irhoud 10, and a mandible, Irhoud 11), and a new date estimate of 315 ka^20^. Well-preserved LMP hominins are more numerous in Eastern Africa. The Singa calvarium from Sudan is dated to 133 ka^21^. In Ethiopia, the Omo Kibish specimens, Omo I and Omo II^22^, are dated to 200 ka^23^, and the three specimens from Herto, which include a complete adult cranium (BOU-VP16/1) and a juvenile calvarium (BOU-VP16/5), with an estimated date of 160 ka^24^. In Kenya, the Guomde calvarium (KNM-ER 3884), which lacks most of the facial and frontal bones^25^, has been dated to 270–300 ka with ɤ-ray spectrometry^26^, while an age of 200–300 ka has been suggested for the nearly complete Eliye Springs skull (KNM-ES 11693^27^) on the basis of its morphology^28^. Further South, a 200–300 ka cranium (LH18^29,30^) was discovered in the Ngaloba Beds at Laetoli (Tanzania), and in South Africa, the site of Florisbad yielded a partially preserved cranium dated to 259 ka^31^. Lastly, the recently discovered remains of *H. naledi*, dated to 236–335 ka^32^, add major complexity to the LMP hominin record of southern Africa.

Even when excluding the *H. naledi* material, African LMP fossils exhibit extremely variable morphologies. The Omo I^22^ and Herto specimens^24^ have a modern-like anatomy that includes the presence of the two cranio-mandibular apomorphies of the species—cranial proportions that result in a tall vault (basi-bregmatic height) and a chin, and are generally considered the earliest undisputed remains of *H. sapiens*^16,17^. All other LMP African fossils show a mosaic of derived and archaic characters. For instance, the Jebel Irhoud remains were originally described as showing strong similarities with Neandertals^33^, while the study of the new Irhoud remains emphasises their affinities with *H. sapiens*, despite the absence of key modern humans apomorphies (i.e., tall and globular vault, and inverted T chin)^18^. The Guomde^25^, Ngaloba^30^, Eliye Springs^27^ and Florisbad^34^ specimens along with Omo II^22^ and possibly the pathological Singa calvarium^35^, have been mostly referred to as ‘archaic *H. sapiens*’, a category grouping isolated fossils with disparate morphologies. This situation challenges any attempt at identifying the evolutionary mechanisms that may explain the morphological pattern in the African LMP fossil record, as well as identifying the ancestral population, or populations, of modern humans.

**Fig. 1:**
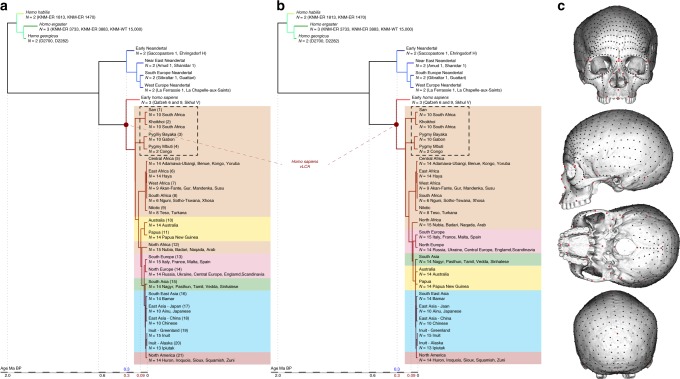
Phylogenetic hypotheses and landmarks distribution on the cranium. **a** and **b** Fully resolved phylogeny of the genus *Homo* according to hypotheses 1 (**a**) and 2 (**b**). **c** Position of the 780 landmarks and semilandmarks used in the study to describe the crania of the phylogeny sample

Here, we use a phylogenetic modelling method^36^ to statistically estimate the full cranial morphology of hypothetical virtual Last Common Ancestors (vLCAs) to all modern humans on the basis of two simplified phylogenies of the genus *Homo* (Fig. 1, Table 1, Supplementary Tables 1 and 2 and Supplementary Fig. 1), and through this virtual LMP African fossil, explore the morphological diversity of the five most complete real African LMP hominins to quantitatively assess how the populations from whom those fossils are drawn may have played a role in the origin of *H. sapiens*.

**Table 1:** Specimens included in the study

Specimens	Chronology^a^	Site	3D^b^	Inst^c^
		Phylogeny sample		
		*H. habilis* sensu lato		
**KNM-ER 1813**	~1.8 Ma	Koobi Fora, Kenya	PH	NMK
**KNM-ER 1470**	~1.8 Ma	Koobi Fora, Kenya	PH	NMK
		*H. ergaster*		
**KNM-ER 3733**	~1.6 Ma	Koobi Fora, Kenya	PH	NMK
**KNM-ER 3883**	~1.6 Ma	Koobi Fora, Kenya	PH	NMK
**KNM-WT 15000**	1.6–1.4 Ma	Nariokotome, Kenya	PH	NMK
		*H. georgicus*		
D2282	~1.77 Ma	Dmanisi, Georgia	PH	IPH
D2700	~1.77 Ma	Dmanisi, Georgia	PH	IPH
		Early Neandertal		
**Saccopastore 1**	130–250 ka	Saccopastore, Italy	PH	US
**Ehringsdorf H**	~200 ka	Ehringsdorf, Germany	PH	IPH
		Near East Neandertal		
Amud 1	50–60 ka	Amud, Israel	OP	DC
Shanidar 1	~45 ka	Shanidar, Irak	PH	MH
		South Europe Neandertal		
**Guattari**	52 ± 12 ka	Monte Circeo, Italy	CT	MP
**Gibraltar 1**	50–60 ka	Forbes’ Quarry, Gibraltar	CT	NHM
		West Europe Neandertal		
**La Ferrassie1**	53–66 ka	La Ferrassie, France	CT	MH
**La Chapelle-aux-Saints**	~50 ka	La Chapelle-aux-Saints, France	CT	MH
		Early *Homo sapiens*		
**Qafzeh 6**	100–130 ka	Qafzeh, Israel	PH	IPH
Qafzeh 9	100–130 ka	Qafzeh, Israel	OP	DC
**Skhūl V**	88–117 ka	Skhūl, Israel	CT	PM
*Holocene*		*Homo sapiens*		
**San (*n*** **=** **10)**	19–20th century	Republic of South Africa	CT/PH	DC/MH
**Khoikhoi (*n*** **=** **10)**	19–20th century	Republic of South Africa	CT/PH	DC/MH
**Bayaka (*n*** **=** **10)**	19–20th century	Central African Republic	PH	MH
**Mbuti (*n*** **=** **2)**	19–20th century	Congo	CT/PH	DC
**Central Africa (*n*** **=** **14)**	19–20th century	Congo–Central Africa–Nigeria	CT	DC
**East Africa (*n*** **=** **14)**	19–20th century	Tanzania	CT	DC
**West Africa (*n*** **=** **9)**	19–20th century	Mali-Ghana-Guinea	CT	DC
**South Africa (*n*** **=** **6)**	19–20th century	Republic of South Africa	CT	DC
**Nilotic (*n*** **=** **8)**	19–20th century	Kenya-Uganda	PH/CT	DC
**Papua (*n*** **=** **14)**	19-20th century	Papua New-Guinea	CT/PH	DC
**Australia (*n*** **=** **14)**	19–20th century	Australia	CT	DC
**North Africa (*n*** **=** **15)**	~5 ka, 19–20th century	Egypt-Sudan	OP/CT	DC
**South Europe (*n*** **=** **15)**	19–20th century	France-Italy-Spain-Malta	CT	DC
**North Europe (*n*** **=** **14)**	19–20th century	Germany-Austria-Russia-Czech Republic-Hungary-Ukraine-Denmark-Sweden-England	CT	DC
**South Asia (*n*** **=** **14)**	19–20th century	India-Pakistan	CT	DC
**South East Asia (*n*** **=** **14)**	19–20th century	Myanmar	CT	DC
**East Asia Japan (*n*** **=** **10)**	19–20th century	Japan	CT/PH	DC/MH/OR
**East Asia China (*n*** **=** **10)**	19–20th century	China	CT	OR/DC
**Inuit Greenland (*n*** **=** **15)**	19–20th century	Denmark	CT	DC
**Inuit Alaska (*n*** **=** **13)**	19–20th century	US-AK	CT	AM
**North America (*n*** **=** **14)**	19–20th century	US-Canada	CT	DC
		LMP sample		
Irhoud 1	~315 ± 34 ka	Jebel Irhoud, Morocco	OP	DC
Omo II	195 ± 95 ka	Omo Kibish, Ethiopia	PH	NMK
**LH18**	200–300 ka	Laetoli, Tanzania	CT	TAZ
**Florisbad**	259 ± 35 ka	Bloemfontein, South Africa	PH	NM
**KNM-ES 11693**	270–300 ka	Eliye Springs, Kenya	PH	NMK

*Note*: Bold types indicate when original specimens were examined

^a^References for chronology can be found in Supplementary Reference

^b^Indicates the scanning method: CT scanner, optical scanner (OP) or photogrammetry (PH)

^c^Indicates where the specimens were scanned (*DC* Duckworth Laboratory, Cambridge, *IPH* Institut de Paléontologie Humaine, Paris, *MH* Musée de l’Homme, Paris, *SU* Museo di Antropologia, Sapienza Università di Roma, *MP *Museo Pigorini, Rome, *NHM* Natural History Museum, London, *NMK* National Museums of Kenya, Nairobi, *NM *National Museum, Bloemfontein, *OR* ORSA database, Penn Museum, *PM* Peabody Museum, Cambridge, *TAZ* National Museum of Tanzania, Dar Es Salaam, *AM* American Museum of Natural History, New York, CT scans from^93^); *Ma* millions of years, *ka* thousands of years

Our main results indicate a complex process for the evolution of *H. sapiens*. While Florisbad shares the most phenotypic affinities with the computed vLCAs, some of the LMP fossils present a different phenotypic profile which supports the recognition of several African hominin populations and lineages. Not all those lineages contributed equally to the origin of *H. sapiens* and our results tend to support the view that *H. sapiens*’ origin may be the result from the coalescence of South and, possibly, East-African source populations. In this scenario, the North-African hominins may represent a population which introgressed into Neandertals during the LMP.

## Results

### Phylogenetic modelling

**Fig. 2:**
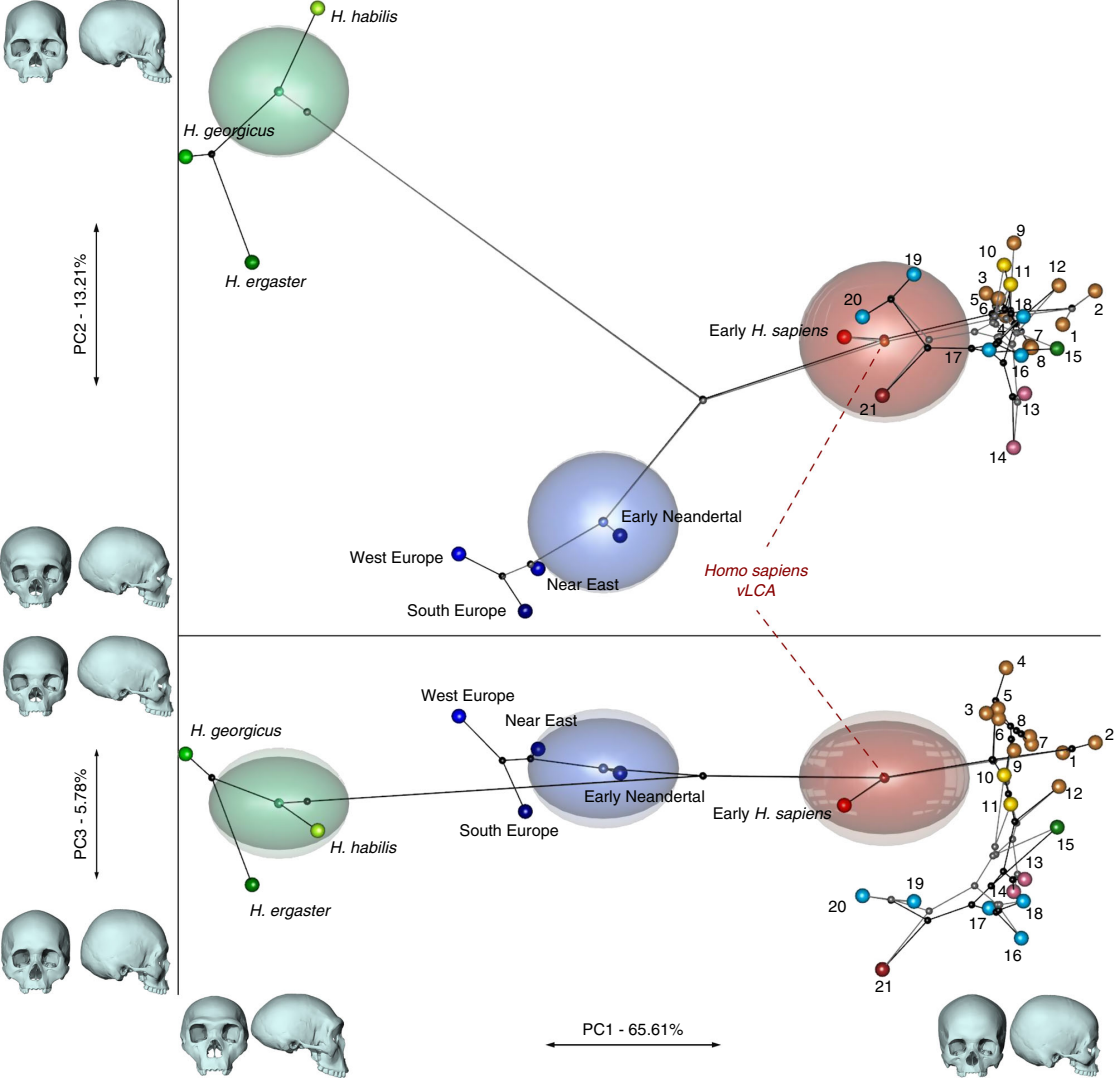
Projection of phylogenetic hypotheses 1 (black) and 2 (grey) into the morphospace. The associated shape deformations are displayed next to each PC. Each node represents estimated ancestors’ shapes along with 95% confidence envelopes. Both trees are similar on PC1 and 2, while PC3 highlights differences between both hypotheses within the modern human clade. Modern human populations as follow: 1 to 9 Sub-Saharan Africa; 10–11 Oceania, 12 North Africa, 13–14 Europe, 15 South Asia, 16 to 20 East Asia, 21 North America (see Fig. 1, Supplementary Fig. 1 and Supplementary Table 1). Source data are provided as a Source Data file

Figure 2 presents the phylo-morphospace and the computed position of the ancestral nodes of hypothesis 1 (black tree) and hypothesis 2 (grey tree) of the *Homo* phylogeny based on 84.6% of the total variation in the data (PC1 to PC3, Supplementary Table 3). Overall, the PCs discriminate accurately the different clades of the phylogenies used. The early *Homo* species (*H. georgicus*, *H. ergaster*, and *H. habilis* s.l.) and the Neandertals have negative values along PC1 (65.6%), contrasting with *H. sapiens* specimens. The associated shape deformation shows a low, elongated calvarium with a strongly projecting face for negative PC1 values, while the positive values are associated with a gracile morphology showing a high, rounded calvarium and an orthognathic face compatible with the San and Khoikhoi populations (#1 and 2). PC2 (13.2%) discriminates mainly the early *Homo* species, which occupy the upper left part of the chart, from modern humans and Neandertals in the lower part of the morphospace. Accordingly, PC2 negative values show a typical Neandertal shape (occipital bun, mid-facial prognathism), whereas PC2 positive values correlate with a small rounded calvarium, and a strongly-projecting face. The phenotypical variation within the modern human cluster is mostly explained by PC3 (5.8%) and follows closely the topology of hypothesis 1. The early *H. sapiens* group is morphologically close to the *H. sapiens* vLCAs and fits within the 95% confidence envelope computed around them. All sub-Saharan populations present positive values (#1 to 9), as well as the Oceanians (#10 and 11) and the North Africans (#12). The Khoisans (#1 and 2) are slightly isolated on PC1, showing the most gracile morphology, while the Pygmies (#3 and 4) have the most positive values on PC3. In the negative values of PC3, South Asians (#15) stand as intermediate between Africans-Oceanians and Eurasians, Europeans (#13 and 14) cluster with South-East Asians (#16) and East-Asians (#17 and 18), and Inuits (#19 and 20) are relatively close to North Americans (#21). The computed *H. sapiens* vLCAs are almost identical for both hypotheses and are more distant from the ancestral node hypothesising the common ancestry between modern humans and Neandertals than the Neandertal ancestor (Fig. 2).

The differences between hypotheses 1 and 2 are subtle. However, the phylogenetic signals^37,38^ computed for hypothesis 1 appear stronger than the ones computed for hypothesis 2 (Supplementary Table 4). This may indicate a better fit of the phenotypic data to phylogenetic hypothesis 1, which includes an earlier wave of dispersal from Africa to Eurasia that preserves a phenotypic signal in Oceania^39^, supported by the similarity of Australians and Papuans with sub-Saharan Africans. The Khoisans and Pygmies branch from the main modern human populations close to the *H. sapiens* LCA^13,40^, but are anatomically derived and gracile^41^. Removing these two groups from the modelling (hypotheses 1b and 2b, Fig. 1) does not alter the coherence of the distribution of the main clades (early *Homo*, Neandertals and *H. sapiens*) or the topologies of both hypotheses in the phylo-morphospace (PC1 to PC3, 85.9%, Supplementary Table 5, Supplementary Fig. 2). However, hypothesis 2b (grey) generates differences impacting on most of the nodes of the tree, resulting in vLCA2b being closer to current modern humans than vLCA1b, and the Australian/Papuan phenotype requiring longer branches between ancestral nodes and terminal taxa to be fitted to the hypothesised topology. This is demonstrated by the computed phylogenetic signals for both hypotheses (Supplementary Table 4).

### Morphologies of the vLCAs

**Fig. 3:**
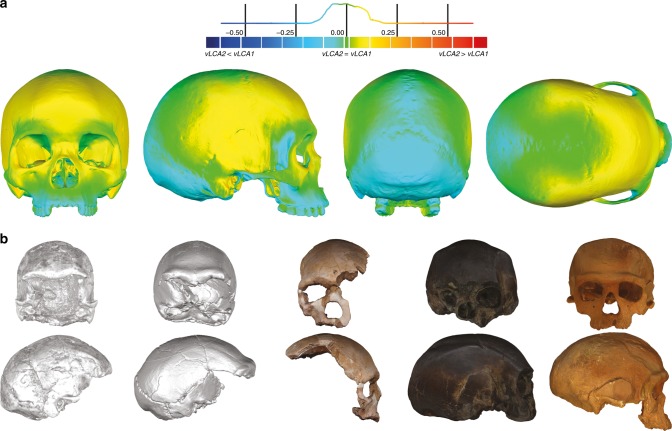
Morphology of the vLCAs and of the LMP fossils. **a** *Norma frontalis*, *lateralis*, *verticalis*, *occipitalis* of vLCA1 when compared to vLCA2 through a surface deviation analysis. The histogram indicates the distribution of the deviation in mm between each vertex of the 3d models. **b** From left to right, *norma frontalis* and *lateralis* of Omo II, LH18, Florisbad, KNM-ES 11693 (the *norma lateralis* view is mirrored), and Irhoud 1. Source data are provided as a Source Data file

The morphologies of vLCA1 and 2 are virtually the same (Fig. 3a, surface deviation <0.16 mm, Supplementary Table 6). Both are relatively gracile in comparison to LMP African fossils; vLCA1 is slightly more robust, with a more receding frontal and a more protruding occipital (Fig. 3b). As expected, the vLCAs present most of the morphological features that would be considered as specific of *H. sapiens*. On the calvarium, the neurocranium is rounded and globular; the frontal bone exhibits a well-developed frontal tuber^42^ and discontinuous supercilliary arches; the parietals show distinct parietal eminences; and the basicranium is markedly flexed. The face is retracted, showing an angulated and forward facing zygomatic^43^, along with a developed maxillary canine fossa with two strongly marked curves (*incurvatio horizontalis* and *sagitallis*^44^). Nevertheless, they also share features with more archaic phenotypes: in *norma lateralis*, the frontal is slightly receding, the brow-ridges are projecting frontward, and separated from the frontal squama by a slight sulcus, the face shows alveolar prognathism, the zygomatic process is aligned with the *crista supramastoidea*^42^, the mastoid processes are weakly developed, and the elongated occipital shows a degree of lambdoid protrusion, which together with a slight depression at obelion, appears like an incipient occipital bun. The last two features could recall the Neandertal morphology^12^. In *norma frontalis*, the antero-posterior border of the maxilla (*incurvatio inframalaris frontalis*) is weakly marked, as observed in *H. heidelbergensis* s.l.^44^, and the interorbital distance is particularly wide^45^. Thus, the vLCAs capture both the uniquely derived aspects of a modern human morphology, and the currently geographically dispersed retention of plesiomorphic characters among different human populations. Hypotheses 1b and 2b, computed in the absence of the Khoisans and Pygmies, generate ancestral shapes which differ only slightly from those based on the original hypotheses (Supplementary Fig. 3a and b). The maximum surface deviation between vLCA1 and 1b and vLCA2 and 2b is nevertheless larger (respectively, <1.00 mm and <2.96 mm, Supplementary Table 6). vLCA1b shows slightly more projecting brow-ridges, but a less prognathic face, while 2b is clearly less robust than vLCA2. When compared to each other the difference is larger (<3.13 mm, Supplementary Table 6) and vLCA2b presents a more gracile morphology (Supplementary Fig. 3c).

### LMP African hominin diversity

**Fig. 4:**
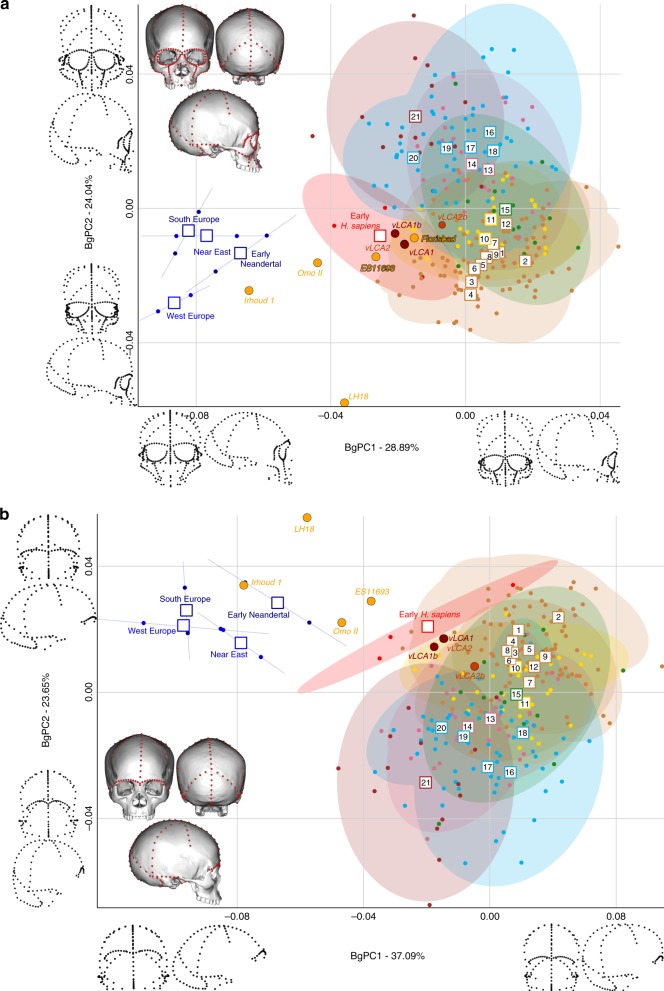
Morphospaces of the bgPCAs for analyses A (**a)** and B (**b)**. The ellipses represent the 90% confidence interval for the estimated distribution of the specimens of each population. The vLCAs are closer in shape to the early *H. sapiens*, as well as the African LMP specimens Flosibad, KNM-ES 11693 and Omo II, while Irhoud 1 is more similar to Neandertals. Modern human populations as follow: 1 to 9 Sub-Saharan Africa; 10–11 Oceania, 12 North Africa, 13–14 Europe, 15 South Asia, 16 to 20 East Asia, 21 North America (see Fig. 1, Supplementary Fig. 1 and Supplementary Table 1). Source data are provided as a Source Data file

**Fig. 5:**
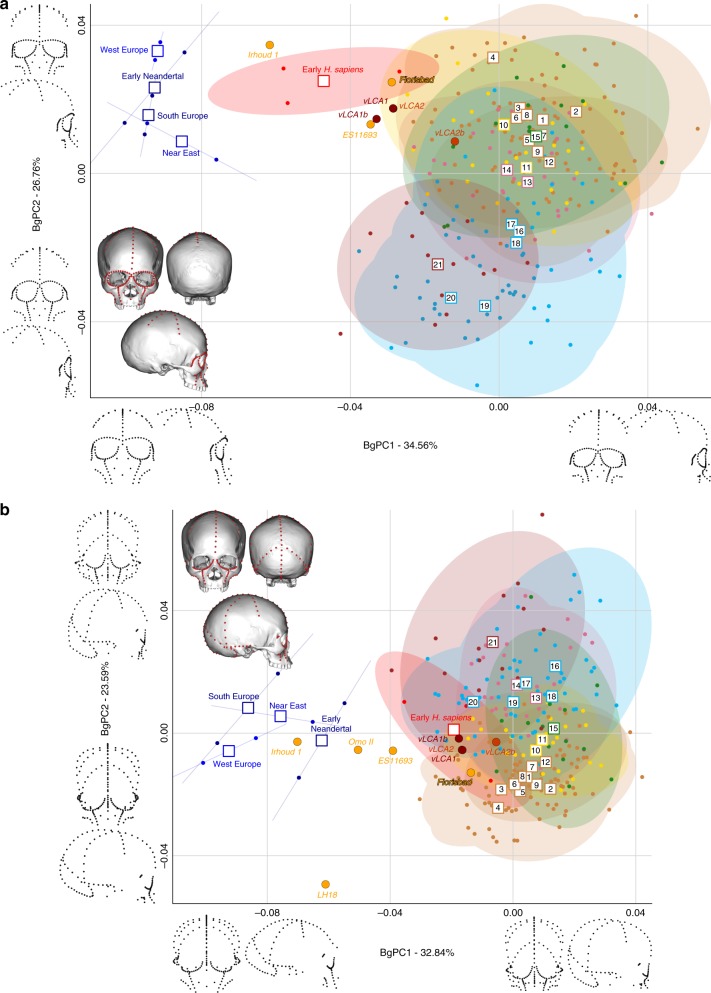
Morphospaces of the bgPCAs for analyses C (**a**) and D (**b**). The ellipses represent the 90% confidence interval for the estimated distribution of the specimens of each population. The vLCAs are closer in shape to the early *H. sapiens*, as well as the African LMP specimens Flosibad, KNM-ES 11693 and Omo II, while Irhoud 1 is more similar to Neandertals. Modern human populations as follow: 1 to 9 Sub-Saharan Africa; 10–11 Oceania, 12 North Africa, 13–14 Europe, 15 South Asia, 16 to 20 East Asia, 21 North America (see Fig. 1, Supplementary Fig. 1 and Supplementary Table 1). Source data are provided as a Source Data file

Figures 4 and 5 show the first two bgPCs of four bgPCAs based on the Procrustes residuals of the individual specimens (to the exclusion of early *Homo*) grouped according to the terminal taxa of the phylogenetic hypotheses, along with the four computed vLCAs and the five LMP fossils. The four bgPCAs were run on different landmark configurations composed of type I homologous landmarks^5^ linked by curves of semilandmarks. The four landmark configurations reflect the preserved data of the LMP fossils (see Methods, Supplementary Table 7 and Supplementary Fig. 6). Figure 4a shows the first two bgPCs (52.9% of variance, Supplementary Table 8) of the bgPCA run on the full skull (Analysis A). BgPC1 discriminates the Neandertals (negative values) from modern humans (positive values), while bgPC2 explains the morphological variation within the modern human sample. The position of the modern human groups reflects their evolutionary history: sub-Saharan Africans (#1 to 9) present negative values, as do the early *H. sapiens* from Qafzeh and Skhūl; Oceanians (#10 and 11), North Africans (#12) and South Asians (#15) cluster around the centre of bgPC2, while all other Eurasians have positive values. The vLCAs cluster around the early *H. sapiens*, but vLCA2b is closer to Oceanians (#10 and 11). The LMP fossils, Florisbad, KNM-ES 11693 and to a lesser extent Omo II are similar to both early *H. sapiens* and the vLCAs, while Irhoud 1 shares more similarities with Neandertals. LH18 presents extreme negative values on bgPC2 due to a receding frontal profile and a short medio-lateral length. The Euclidean distances between group means (Supplementary Data 1) confirm these patterns: vLCAs 1, 2 and 1b are closer to early *H. sapiens* (respectively, 0.0254, 0.0255 and 0.0230), while vLCA2b is closer to Australians (0.0240). Florisbad is the African LMP specimen which resembles the vLCAs the most, particularly vLCA1 and 2 (0.0419), while Irhoud 1 is the only LMP specimen sharing the most similarities with Neandertals (South Europe Neandertals: 0.0617). Figure 4b shows the two first bgPCs (60.7%, Supplementary Table 9) of the bgPCA run only on the calvarium and excluding Florisbad (Analysis B, Supplementary Table 7 and Supplementary Fig. 6). The morphospace is similar to analysis A. VLCAs 1, 2 and 1b are similar to early *H. sapiens*, while vLCA2b is closer to current populations. Omo II and KNM-ES 11693 show phenotypic affinities with early *H. sapiens* and early Neandertals, while Irhoud 1’s morphology shares similarities with Neandertals. VLCAs 1, 2 and 1b present the lowest Euclidean distance (Supplementary Data 2) to early *H. sapiens* (respectively, 0.0233, 0.0235 and 0.0214), while vLCA2b is closer to East Africans (0.0202). Within the LMP fossils, Omo II shares the most similarities with the vLCAs (0.0672 with vLCA1b) but is closer to early *H. sapiens* (0.0665). Irhoud 1 shortest Euclidean distance is to South Europe Neandertals (0.0395).

Figure 5 displays the last two bgPCAs run on the preserved landmark configuration of Florisbad (Analysis C, Fig. 5a) and KNM-ES 11693 (Analysis D, Fig. 5b). Omo II and LH18 are not included in analysis C. Both morphospaces preserve the same overall distribution of points. However, in analysis C’s morphospace (61.3%, Supplementary Table 10), early *H. sapiens* are positioned between modern humans and the Neandertals. VLCAs 1, 1b and 2 are situated between current modern and fossil *H. sapiens*, and share strong similarities with Florisbad and KNM-ES 11693. Contrary to analyses A and B, Irhoud 1 is closer to early *H. sapiens* and is overall less similar to Neandertals (Fig. 5a). This is expected, as analysis C focuses mostly on facial morphology which is more modern in Irhoud 1 than its vault^18^. The Euclidean distances (Supplementary Data 3) underline this pattern of affinities: vLCAs 1, 2 and 1b are closer to the early *H. sapiens*, while vLCA2b is closer to Australians. Florisbad is the most similar to the vLCAs (0.0502 with vLCA2) and Irhoud 1 is closest to the early *H. sapiens* (0.0480). Finally, Fig. 5b presents the morphospace (56.4%, Supplementary Table 11) of analysis D. The exclusion of the missing landmarks of KNM-ES 11693 modifies slightly the relative phenotypic affinities, with early *H. sapiens* being more closely grouped with current modern human populations. The vLCAs cluster close to the centre of early *H. sapiens* variation, with the exception of vLCA2b (Fig. 5b). Florisbad and KNM-ES 11693 are the closest to both the *H. sapiens* fossils and the vLCAs. Omo II occupies a central position between modern and Neandertal populations, while Irhoud 1 presents strong affinities to Neandertals. Finally, the associated Euclidean distances (Supplementary Data 4) underline the strong similarities between vLCAs 1, 2 and 1b and early modern humans (respectively, 0.0211, 0.0212 and 0.0187), while vLCA2b is closer to Papuans (0.0214). Florisbad is the closest African LMP specimen to the vLCAs (0.0364), followed by Omo II (0.0605) and KNM-ES 11693 (0.0678). Irhoud 1, as in analysis A and B, is closest to the South European Neandertals (0.0476).

The Procrustes distances-based boxplots (Supplementary Figs. 4 and 5) present the similarities of the vLCAs to the African LMP, Neandertal and *H. sapiens* specimens for analyses A B, C and D.

In analysis A (Supplementary Fig. 4a and b), the vLCAs are closer to the modern human groups, especially to early *H. sapiens*, with which vLCAs 1, 2 and 1b share the shortest median distance. These three vLCAs are even closer to Florisbad, with Procrustes distances shorter than the median Procrustes distance to any other group. LH18, KNM-ES 11693, Irhoud 1 and Omo II Procrustes distances to the vLCAs are larger. vLCA2b shares more similarities with current modern humans. The overall similarity pattern with the vLCAs is comparable in analysis B (Supplementary Fig. 4c and d). However, in the absence of Florisbad, none of the African LMP fossils share strong similarities with the vLCAs: the shortest distance is to Omo II, but this is higher than any of the median distances to modern humans. Analysis C (Supplementary Fig. 5a and b) is comparable to analysis A: vLCAs 1, 2 and 1b, present a similar distance to Florisbad than to the median of early *H. sapiens*, and vLCA2b is closer to recent modern humans. KNM-ES 11693 and Irhoud 1 present larger Procrustes distances to vLCAs 1, 2 and 1b than their median distances to modern humans. Finally, analysis D (Supplementary Fig. 5c and d) depicts a different picture, as the vLCAs are closer to modern humans than to the African LMP fossils (KNM-ES 11693, LH18, Omo II and Irhoud 1) to the exception of Florisbad.

## Discussion

The methodological approaches used in the present study have inherent uncertainties—biases from using different means and/or operators^46–48^ to obtain 3D data, number of variables versus the number of specimens, sliding of semilandmarks^49–51^, and sampling error to obtain mean population shapes^52^. To minimise this, the data were collected by a single operator; we reduced the number of variables by using PCs^53^ instead of aligned 3D coordinates throughout the study, and test models were run on non-slid semilandmarks, as well as subsamples of landmarks and semilandmarks (Supplementary Figs. 7–10). Supplementary Fig. 8 shows that the number of landmarks and the use of slid semilandmarks^50^ do not impact the reliability of the results. While sample size remains limited to the available fossil sample, sampling error can be assessed in the bgPCA analyses where specimens were analysed individually (Figs. 4 and 5, Supplementary Data 1–4). Finally, the modelling approach used here computes ancestral shapes which are weighted averages derived from the terminal taxa of a phylogenetic hypothesis^54^ of given topology using a Brownian motion model for evolution that mostly estimates drift. Those ancestral estimations are not intended to accurately represent evolution, but to act as tools to study the African LMP hominin fossil record in order to bring new insights into the origin of *H. sapiens*.

Our findings show that, first, the phenotypic data presented in this study relate closely to the genetic history^39,55^ of the considered populations. This is illustrated by the phylo-morphospace based on mean population shape data (Fig. 2), but also by the distribution of individual specimens’ shapes (Figs. 4 and 5). In addition, hypothesis 1, which takes into account an early out-of-Africa which preserves a phenotypic signal among the people of Sahul^39^, and hypothesis 2, which follows a more classic out-of-Africa event^55^, led to the computation of ancestral morphologies which are virtually undistinguishable (Fig. 3a, Supplementary Table 6). Yet, the topology of the phylogenetic tree of hypothesis 1 fits more closely the phenotypic variation of the data, as exemplified by the phylogenetic signal computed for the different phylogenies (Supplementary Table 4). The exclusion of the Khoisans and Pygmies has little impact on the first hypothesis, as the phenotype of vLCA1 and 1b remains comparable (Supplementary Fig. 3). Hypothesis 2 is more affected by this change, resulting in a very gracile morphotype (Supplementary Fig. 3) and lower values for the phylogenetic signal metrics (Supplementary Table 4). Therefore, the scenario simulated in hypothesis 1 appears to be slightly better supported by the phenotypic data presented here, which is congruent with recent genomic results^14,39^.

Second, the vLCAs’ morphologies are gracile and modern. The derived cranial features of *H. sapiens* are fully displayed in the vLCAs—a domed neurocranium, a reduced face and a marked basicranial flexion^17^, and only partly balanced by more archaic features (i.e., projecting brow-ridges, marked alveolar prognathism, weakly developed mastoid processes, elongated occipital bone, weakly marked *incurvatio inframalaris frontalis*, and wide interorbital distance; see refs. ^12,42,44,45^). One explanation for this gracile morphology can be found in the structure of the phylogenetic hypotheses. Under a Brownian Motion model of evolution, branch length contributes significantly to the ancestral reconstruction at the nodes^54^. Terminal taxa with long branch length will contribute more to the ancestral estimation. In the present hypotheses, the strongest influence on the ancestral reconstruction should be the Khoisans and Pygmies and, to a lesser extent, early *H. sapiens*. The Khoisans and Pygmies branch out very early from the *H. sapiens* stem lineage^13,40^, which may give an ‘archaic’ weight to their particularly gracile morphologies^41^. The derived morphologies of these populations, partly due to adaptation to the environment^56^, should be different from their ancestors’ phenotypes. The shape of the vLCAs may hence be influenced by this Khoisan/Pygmy morphological pattern by overestimating the antiquity of this gracile phenotype. This effect may only be partly balanced by the comparatively large and robust individuals (Qafzeh 6 and 9, and Skhūl V) of the early *H. sapiens* terminal taxon, since they are chronologically older than extant human populations and have a consequently shorter branch length (i.e., ~100 ka^19,57^). However, the early *H. sapiens* taxon plots within the 95% confidence envelope of the vLCAs in the phylo-morphospaces (Fig. 2 and Supplementary Fig. 2). In addition, the divergent morphologies (Supplementary Figs. 2 and 3) estimated from the b hypotheses reinforce this idea: the least plausible phylogeny (hypothesis 2b) yields the least plausible ancestral estimation for modern humans (vLCA2b). Thus, the gracile morphology of the computed vLCAs, while influenced by the theoretical framework of the modelling approach used here, appears to be more than just an artefact of the derivation of the ancestral morphologies from the extant descendants.

Despite the aforementioned uncertainties, and given the phenotypic affinities displayed by the LMP African hominins, a palaeoanthropological interpretation of those results is possible. The rapid fixation of the derived cranial traits shared by all recent *H. sapiens* with variable expression of robusticity traits^58^ could explain the phenotypes of the vLCAs. This could possibly have occurred through a process of localised drift during the very late Middle Pleistocene leading to population structuring^16^. The African LMP fossil mosaic morphologies combine archaic and modern characters, and the first occurrence of a full modern morphotype is not documented before Omo I (195 ka) and BOU-VP16/1 specimen from Herto (160 ka). None of the other LMP fossils shows the combined expression of modern human derived traits, while the computed vLCAs are modern humans and the universal cranial characters shared by all members of the species are present. This could support a rapid appearance of *H. sapiens*, consistent with the punctuated equilibrium theory^59^, however there is no evidence for a long and stable period of stasis in the Middle Pleistocene fossil record. On the contrary, current evidence suggests a dynamic biogeographic period driven by pronounced shifts in aridity and temperature^60^, during which drift and selection would have acted to create diversity at population and potentially species levels.

This scenario is consistent with the high level of phenotypic diversity in the LMP African hominin record. The results presented here highlight this diversity and allow to hypothesise the existence of different LMP morphotypes in Africa reflecting a structured population history (Fig. 4b). Three broad morphological patterns may be identified in the LMP fossil record: one represented by the Eastern African fossil LH18, the African LMP fossil sharing the least affinities with modern humans and the computed vLCAs (although some distortions^61^ on the calvarium may partly explain these phenotypic affinities); another exemplified by the North African fossil Irhoud 1, whose morphology is intermediate between modern humans and the Neandertals; and a possible third morph, represented by the South African fossil of Florisbad, and, to a lesser extent by the East African fossils KNM-ES 11693 and Omo II. Those three specimens present the shortest Euclidean distances to modern humans in all four analyses when the vLCAs are not taken into consideration (Supplementary Data 1–4). Indeed, the second shortest Euclidean distances observed is between Florisbad and early *H. sapiens*, between KNM-ES 11963 and the San, and between Omo II and early *H. sapiens*, and for analysis D with Alaskan Inuit (Supplementary Data 1–4). On the other hand, shortest Euclidean distance between Irhoud 1 and other groups is always to the South European Neandertals, except for analysis B where it is with early *H. sapiens*. Analysis B focuses mostly on the face which is where modern human-like traits have been identified in Irhoud 1^18^. Finally, Florisbad is the LMP fossil most similar to the vLCAs, as exemplified by the Euclidean (Supplementary Data 1, 3, and 4) and Procrustes distances (Supplementary Figs. 4 and 5), and by the presence of a well-defined canine fossa, a fully formed *tuber frontale* and weakly projecting brow-ridges (Fig. 3b).

These results resonate with the age of the South African *H. naledi* remains^4^, a small-brained hominin with an unusual mosaic of ancient and modern traits, dated to the beginning of the LMP (i.e., 335–236 ka^32^). Both the discovery of an LMP ‘archaic’ looking *Homo* species and the results of the present study underline the complexity of the morphological variation within the genus *Homo* during the African LMP. As well as a high degree of population structure within the *sapiens* evolving lineage as suggested by our analyses, this complexity may encompass the presence of different *Homo* lineages in Africa. While some populations descending from an African *H. heidelbergensis* lineage may have coalesced to form *H. sapiens* following the African Multiregionalism hypothesis^9^, it is likely that some LMP African fossils were not associated with any population ancestral to *H. sapiens*. We should thus expect to find LMP fossils in Africa that were members of chronologically and geographically overlapping side branches of the Pleistocene human evolution tree, as the *H. naledi* remains seem to be^4^, reflecting different time-depths of lineage formation and extent of adaptive differences. The eventual loss of these side branches could be the result of the cumulative effect of differential resilience to both climate change and inter-group competition. While the LMP fossil and archaeological record is at present insufficient to build a coherent model of diachronic change in population distributions, it could suggest a multi-phase^16^ process for the evolution of modern humans and their diversity. After the origin of the species in the late Middle Pleistocene, a second phase characterised by the successful expansion of modern human populations^16^ may have started as early as 194 ka^3^ but is clearly established by 130 ka, as documented by the increase in number of archaeological sites^62^, and the presence of a modern human population in the Levant^3,43^. The *H. sapiens* fossils from East Asia^63^, whose lower chronological boundary may extend to 120 ka^64^, may have been part of this earlier expansion phase. Genomic analysis offer conflicting evidence regarding such an early expansion of modern humans in Eurasia. The reported introgression of modern human DNA into Neandertals who lived *ca*. 100,000 years ago in the Altai Mountains^65^ would suggest that African populations had indeed expanded beyond the Levant during the last interglacial, as do some of the results based on the genotypic variation of Australians and Papuans today^14,39,55^. The contribution of this out-of-Africa dispersal during Marine Isotope Stage (MIS) 5 to the origin of current human diversity in Eurasia remains unclear. Nevertheless, other hominin populations and species (e.g., *naledi*) in Africa seem to have largely disappeared by MIS5, possibly due to the harsh climatic conditions during MIS6^66^, which may have triggered local extinction events, and to competition with expanding groups of *H. sapiens* during the early phases of MIS5.

Aside from the complexity of the potential interactions among LMP African hominins, what can the present study tell us about the geographic origin of the first *H. sapiens*? Current genomic data point towards a southern^67^ or an eastern African origin^68^, or one across an east-south African cline;^69^ palaeoanthropology suggests a northern^18,70^, or eastern African origin^22,24,62^. Our results tend to support a complex evolutionary pattern that may have involved different source populations, possibly including south and east African groups as it has been recently advocated through genomics^69^. Both the Southern African fossil Florisbad, and the Eastern African specimens KNM-ES 11693 and Omo II show similarities with the vLCAs and early *H. sapiens*. A northern African origin is less likely^18^, as Irhoud 1 displays a different affinity pattern, making it morphologically closer to the Neandertals. The newly described specimens from Jebel Irhoud brought new information regarding the morphology of LMP North African hominins. The mandible Irhoud 11 shows some derived features (i.e., forward increase in mandibular body height and narrow section), but lacks a full modern human inverted T chin^18^ as was noted before in the juvenile Irhoud 3 specimen^71^. The fragmentary upper face (Irhoud 10), is morphologically similar to the upper face of Irhoud 1^18^, which, in the latter specimen falls within, but at the edge of the distribution of modern human facial morphologies^36^, or, when analysed as an integrated part of the cranium in the present study, shares more similarities with a possible modern human and Neandertal LCA than with the computed *H. sapiens* vLCAs. An alternative hypothesis regarding the North-African population represented by the Jebel Irhoud fossils could build upon recent genomic results suggesting that the ancestral Neandertal mitochondrial DNA (mtDNA), shared with the Denisova^72^ and Sima de los Huesos^73^ fossils, was replaced by an African mtDNA between 460 and 219 ka^15^. Considering the phenotypic affinities of Irhoud 1 with both Neandertals and early *H. sapiens*, it is possible that the Irhoud fossils represent local descendants of an African population that dispersed out of Africa during a Green Sahara event associated with MIS9, thus related to the African populations which introgressed into the European lineage at this time, and hence contributing to the evolution of the ‘classic’ Neandertals.

Following from these results, it is possible to tentatively draw a framework for the origin of *H. sapiens*. The speciation process appears to have been complex, going through different phases^16^ that may not have contributed to the genetic and phenotypic structure of current modern human populations. A first stage of phenotypic diversification, from 350 to 200 ka, may have happened locally with different contemporary populations forming local morphs of pre-*H. sapiens* groups as they are represented in the LMP fossil record. This phase may have been followed by a period of fragmentation and differential expansion of populations leading to hybridisation and coalescence of groups, which could have resulted in the emergence of morphologically derived populations of anatomically modern humans between 200 to 100 ka, as exemplified by the fossils from Herto, Skhūl and Qafzeh. Nevertheless, our results suggest that it is unlikely that all LMP local populations would have contributed equally, or at all, to the lineage that gave rise to the population ancestral to *H. sapiens*; local extinctions and founder effects would have shaped considerably the emergence of anatomically modern humans^16^. The morphology of the vLCAs computed in the present study appears to be closer to this last phase (i.e., 200–100 ka) than to the former one. This may indicate that chronologically older fossils of anatomically modern *H. sapiens*, representing populations which outlived most of the LMP hominin groups, are yet to be found.

## Methods

### Materials

We used a sample of 263 3D crania (see below and Table 1, Supplementary Table 1, Supplementary Fig. 1) to produce mean populations shapes which serve as terminal taxa of the phylogeny of the genus *Homo* (Fig. 1a, b): seven early Pleistocene specimens representing the species *H. habilis*, *H. ergaster*, and *H. georgicus;* eight Neandertals from amongst different geographical populations (early, Near-East, South-Europe and West-Europe Neandertals), and three early *H. sapiens* fossils (Qafzeh 6 and 9, Skhūl V). 245 extant modern humans represent current *H. sapiens* groups and are organised through 21 populations (i.e., ten Africans, eight Eurasians, two Inuits and a North American group, see Supplementary Table 1 and Supplementary Fig. 1). The five African LMP targeted fossils (Table 1, Fig. 3) are from North Africa (Irhoud 1), from East Africa (KNM-ES 11693, LH18 and Omo II), and from South Africa (Florisbad), and they were not included in the estimation of the *H. sapiens* vLCAs. These specimens are among the few fossils that could represent the ancestral population to all modern humans: besides originating in Africa, their estimated dates fit within the estimated timeframe put forward by palaeoanthropological and genomic studies for the origin of *H. sapiens*^9,69^, as well as with the most recent date estimates based on the study of Khoisan ancient genomes suggesting that the last genetic common ancestral population of all living humans may have lived more than 260,000 years ago^69^. We did not include the fossils of Guomde^25^ and Omo I^22^ due to their preservation state, or Singa due to its pathological condition^35^. We did not have access to the BOU-VP16/1 specimen.

### 3D models building

The 3D models were obtained following three procedures (Table 1): (1) medical computed tomographic scans (voxel size between 0.449219 and 0.488281 mm) processed in Amira (v5.5, FEI); (2) photogrammetry using the Photoscan software (Agisoft, v1.2.6); and (3) 3D surface scans using an optical scanner (HDI Advance, 45 µ accuracy, LMI) and the FlexScan 3D software (v.3.3, LMI). The accuracy of photogrammetry is sufficiently good to produce reliable 3D Models with an error margin similar to that of laser surface scanning^47^. Comparison of 3D models obtained from medical computed tomographic scans and laser surface scanning also show that both methods produce comparable quality models^48^. The resulting models (on average 1.5 million vertices), from both the phylogeny and LMP samples, are described by 780 landmarks (Landmark software, IDAV^74^) among which 724 are semilandmarks (116 on curves located on the face and 608 on surfaces located on the calvaria) which are allowed to slide^5,75^ (Fig. 1b). To estimate the impact of sliding semilandmarks on the present analysis, a test was run on hypothesis 1 comparing results from non-slid semilandmarks coordinates to the results presented in this manuscript. Differences between both analyses are small (Analysis E, Supplementary Figs. 7 and 8, Supplementary Tables 12–14). Large number of variables may have effects on some analyses^49^. To verify this impact on the present analysis, two tests were run on hypothesis 1, and two vLCAs (vLCA1Sub and vLCALd) were computed based on a subsample of 239 semilandmarks and on 53 landmarks only. The difference in the morphology of the vLCAs is small (Analysis F, Supplementary Figs. 8–10, Supplementary Tables 14–17). Sliding of semilandmarks minimises shape differences between each specimen and the average shape in the sample (i.e., variation due to arbitrary spacing of the points coordinates)^50^. The approach modifies slightly the raw data coordinates and has been debated in the past^50,51^. Missing landmarks are estimated by mirroring existing landmarks onto the other side, and when this is not possible, the few remaining missing landmarks were estimated by thin-plate-spline interpolation (i.e., TPS^76^). Florisbad, Omo II and LH18 required major reconstruction to be aligned (GPA) with the vLCAs and the phylogeny sample. However, the analysis and comparison of their morphology is based on the preserved anatomical configuration of each specimen. To correct for bilateral asymmetry, we used the symmetric component from the Generalised Procrustes Analysis (i.e., GPA^77^) in all subsequent analyses. The data collection, photogrammetry, surface scanning, and segmentation for 3D model building, along with collection of landmarks, were done by one of us (AM).

### Phylogenetic modelling

To compute the morphology of the vLCAs, we use two hypotheses representing fully resolved phylogenies of the genus *Homo* (Fig. 1a, b). Both hypotheses are based on the same general topology: three species (*H. habilis*: KNM-ER 1813 and 1470, *H. ergaster*: KNM-ER 3733, KNM-ER 3883 and KNM-WT 15000, and *H. georgicus*: D2282 and D2700) compose the outgroup of the two sister taxa, Neandertals (Early (Near-East (South Europe, West Europe))), and modern humans. The Neandertal clade is built to reflect possible sub-groups in the population:^78^ ‘classic’ Western Neandertals, more derived than southern, Near-Eastern and Early Neandertal individuals. The modern human clade is a simplification of our evolutionary history:^11^ the Qafzeh and Skhūl specimens being a sister group to extant *H. sapiens*^43^, who are arranged following their relative genetic relationships (see Supplementary Table 2 and refs. ^14,39,55^). The terminal taxa are positioned in relation to their chronology; the nodes of the phylogeny reflect consensual chronologies based on genetic and palaeoanthropological data^8,36,55^, and the position of the early *Homo* outgroup is based on extensive palaeoanthropological studies^2^. The common ancestry between modern humans and Neandertals is positioned at 600 ka, following suggestions from latest genomic estimates^8^ and from previous phylogenetic modelling results;^36^ the Neandertal clade is rooted at around 300 ka and the modern human clade at 305 ka^69^ (Fig. 1 and Supplementary Table 2). Both hypotheses place the African populations at the base of the clade. Hypothesis 1 takes into account an extinct early out-of-Africa which can be detected in current Oceanian populations’ genomes^39^. This migration event, which was followed by a later out-of-Africa, left a significant but small signature in the current Sahul populations along with other signatures from admixture with Neandertal and/or Denisovan populations^39^. In order to simulate this complex scenario in hypothesis 1, the Australian and Papuan branches split from the African clade at 90 ka corresponding to the median genetic split time between West Africans and Papuans calculated by Pagani and colleagues^39^ (Fig. 1a). Hypothesis 2 ignores this possible extinct out-of-Africa, and follows a more traditional topology^55^, with non-African populations splitting from the Africans at 75 ka (Fig. 1b). Two alternative hypotheses (i.e., hypotheses 1b and 2b) removing the Khoisan and Pygmy populations (Fig. 1) from the phylogenies were also tested. The continuous variables used to describe the terminal taxa are principal components (i.e., PCs) representing the mean shape variables for each population obtained after performing a GPA and a Principal Component Analysis on the specimens’ landmark sets. The ancestral shapes are computed for each of the mathematically uncorrelated PCs using maximum likelihood^79^ within a model of evolutionary change assuming random walks in continuous time (i.e., Brownian motion model^80^) which mostly approximates genetic drift but can also reflect adaptive evolutionary changes;^81^ however, genetic drift seems to explain most of the cranial morphological divergence observed between some hominin populations^82^. The use of PCs to perform cladistics analyses^83^ has been criticised^84^, but PCs are considered more suitable for maximum likelihood based analyses^85^. Maximum likelihood reconstructs the ancestral states to maximise the probability of the states observed among the known taxa of a given hypothetical phylogeny (i.e., topology, branch length) following a statistical evolutionary divergence model^79^ (i.e., Brownian motion model^80^). The maximum likelihood approach computes for each node of the phylogeny the most likely ancestral shape along with a 95% confidence envelope (Fig. 2). The confidence envelope is a quantification of the uncertainty based on a standard probabilistic measure that depends on the rate of variance of the characters, the length of the branches, and the topology of the phylogeny. In the case of a deep ancestry between two sister taxa, the uncertainty is larger, a more recent common ancestry will present less uncertainty^54^. The ancestral PCs are rotated back into the landmark space^86^ giving a set of coordinates in which a unique modern human skull (specimen Kh-1739; see Supplementary Table 1) is warped (TPS warping^76^) to produce the different fully rendered 3D vLCAs (see ref. ^36^ and Fig. 3). Finally, to assess the correlation between each phylogenies and the data imputed, we computed their phylogenetic signal for the PCs and the aligned 3D coordinates using a multivariate K statistic^37,38^.

### Surface spectrum deviation

To assess the morphological differences between the vLCAs obtained through the 4 models tested here (i.e., hypotheses 1, 2, 1b and 2b), we used a surface spectrum deviation method that quantifies the surface deviation between a reference and a test specimen. The ancestral landmarks are aligned (GPA) and a 3D model of a modern human (Kh-1739) is warped into the configurations. The difference in shape can then be explored through a colour-coded spectrum, which gives a colour to each of the vertices of the 3D model according to the deviation, measured in millimetre, from the reference. The deviation can be visualised as histograms, showing the distribution of the test specimen’s vertices according to their distance to the reference specimen’s vertices (Fig. 3 and Supplementary Fig. 3). Figure 3a presents the deviation of vLCA2 (test) against vLCA1 (reference). Supplementary Fig. 3 presents the surface deviation of vLCA1b vs 1 (A), vLCA2b vs 2 (B), and vLCA2b vs 1b (C). Finally, for each comparison we calculate the maximum positive and negative distances, the average positive and negative distances, and the standard deviation (Supplementary Table 6).

### 3D geometric morphometrics

To assess the pattern of morphological variation of the vLCAs and of the LMP fossils, we ran four analyses using sub-samples of the landmark configuration used to compute the phylogenetic models. The first analysis uses 255 landmarks and semilandmarks distributed on the full skull along the cranial sutures (i.e., bregma-stephanion, bregma-lambda, lambda-asterion) along with additional curves joining craniometric points (i.e., bregma-asterion, asterion-porion, asterion-opsithion, opisthion-lambda, frontomalare orbitale-zygoorbitale, zygoorbitale-canin, zygoorbitale-maxillofrontale, frontomalare temporale-jugale, zygotemporale-lateral M1, glabella-frontozygomatic, Analysis A, Supplementary Fig. 6a); the second is based on the preservation state of Omo II and LH18 and uses only 148 landmarks and semilandmarks on the calvarium (Analysis B, Supplementary Fig. 6b); the third is based on the preservation state of Florisbad and uses 112 landmarks and semilandmarks on the calvarium and face (Analysis C, Supplementary Fig. 6c); and the last analysis is based on the preservation state of KNM-ES 11693 and uses 181 landmarks and semilandmarks on the calvarium and face (Analysis D, Supplementary Fig. 6d). Due to their state of preservation, Omo II and LH18 were discarded from analysis C and Florisbad was discarded from analysis B (Supplementary Table 7). For each analysis, the Neandertal and modern human specimens, used to generate the models, were aligned (GPA) individually along with the vLCAs and LMP fossils. The Procrustes distances from the GPA were examined through boxplots (Supplementary Figs. 4 and 5) and the Procrustes residuals were used to compute four between group Principal Components Analyses (i.e., bgPCA, Figs. 4 and 5) grouping the specimens according to their population, to the exception of the vLCAs and LMP fossils which were not given a group. The Euclidean distances between mean groups were considered to clarify the phenetic relationships of the vLCAs with LMP fossils and the hominin groups (Supplementary Data 1–4).

We used Geomagic Studio v.2013.0.1 to perform the surface deviation spectrum analysis and R for all other analyses (Morpho v2.1;^87^ geomorph v2.1.2;^88^ ape v3.2;^89^ phytools v0.4–3.1^90^, Ade4 v1.7–4^91^, phylocurve v2.0.9^92^).

### Reporting summary

Further information on research design is available in the Nature Research Reporting Summary linked to this article.

## Supplementary information

Supplementary Info

Reporting Summary

Description of Additional Supplementary Files

Supplementary Data 1

Supplementary Data 2

Supplementary Data 3

Supplementary Data 4

Supplementary Data 5

Supplementary Data 6

Supplementary Data 7

Supplementary Data 8

Supplementary Data 9

Supplementary Data 10

Supplementary Data 11

Supplementary Data 12

Supplementary Data 13

Supplementary Data 14

Supplementary Data 15

Supplementary Data 16

Supplementary Data 17

Supplementary Data 18

Source Data

## Data Availability

The 3D model of the vLCAs are available in Supplementary Data 5–11 along with the 3D models showing the deviation patterns between the vLCAs (Supplementary Data 12–18). In order to visualise properly Supplementary Data 5–18, please use the open source Meshlab software or use the R software (see Description of Additional Supplementary Files). The 3D geometric morphometric data (i.e., landmarks coordinates) are available in the Source Data File.
