# Supplementary material for: Deciphering African late middle Pleistocene hominin diversity and the origin of our species
Source: Nat Commun. 2019 Sep 10;10:3406. doi: 10.1038/s41467-019-11213-w (PMC6736881; doi:10.1038/s41467-019-11213-w)
Supplement: Supplementary file 1 — Supplementary Info [file 41467_2019_11213_MOESM1_ESM.pdf]

Supplementary Information

Deciphering African Late Middle Pleistocene hominin diversity and the origin of our Species

Mounier and Mirazón Lahr

Table of Contents

Supplementary Tables 3

Supplementary Table 1. Description of the specimens included in the study[a]. 3

Supplementary Table 2. Description of the phylogenetic hypotheses, showing the topology of the trees along with associated split times and references. 9

Supplementary Table 3. Description of the PCs from the aligned tree data for Hypotheses 1 and 2 12

Supplementary Table 4. Values for the phylogenetic signal K_{mult} (i.e. multivariate K-statistics from Blomberg's K[106]) for hypotheses 1, 2, 1b and 2b. 13

Supplementary Table 5. Description of the PCs from the aligned tree data for Hypotheses 1b and 2b 14

Supplementary Table 6. Maximum, average and standard deviation of the surface deviations between the different vLCAs (mm). 15

Supplementary Table 7. African LMP specimens' percentage of preserved landmarks for GMM analyses 15

Supplementary Table 8. Description of the between group PCs – Analysis A 16

Supplementary Table 9. Description of the between group PCs – Analysis B 17

Supplementary Table 10. Description of the between group PCs – Analysis C 18

Supplementary Table 11. Description of the between group PCs – Analysis D 19

Supplementary Table 12. Description of the PCs from the aligned tree data for Hypothesis 1 using row data before sliding and data after sliding semilandmarks 20

Supplementary Table 13. Description of the between group PCs using a sub-sample of 239 landmarks 22

Supplementary Table 14. Values for the phylogenetic signal K_{mult} (i.e. multivariate extension of Blomberg's K-statistics[106]) of phylogenetic hypotheses 1, 1NS, 1Sub and 1Ld. 23

Supplementary Table 15. Description of the between group PCs using a subsample of 53 landmarks 24

Supplementary Table 16. Description of the PCs from the aligned tree data for Hypothesis 1 using a sub-sample of 239 landmarks and semilandmarks 25

Supplementary Table 17. Description of the PCs from the aligned tree data for Hypothesis 1 using a sub-sample of 53 landmarks. 26

Supplementary Figures 27

Supplementary Figure 1. World map showing the geographical and ethnical origin of the sampled population. 28

Supplementary Figure 2. Projection of the phylogenies 1b (black) and 2b (grey) in the morphospace. 29

Supplementary Figure 3. Surface deviation analyses comparing the vLCAs' morphologies. 30

Supplementary Figure 4. Box-and-whisker plots showing the Procrustes distances between the vLCAs and the comparative sample for analyses A and B. 32

Supplementary Figure 5. Box-and-whisker plots showing the Procrustes distances between the vLCAs and the comparative sample for analyses C and D. 34

Supplementary Figure 6. Landmarks configurations used to run the GPAs and bgPCAs. 35

Supplementary Figure 7. Results from the modelling based on hypotheses 1 and 1NS. 36

Supplementary Figure 8. Morphospaces of bgPCAs for analyses E and F. 39

Supplementary Figure 9. Results from the modelling based on hypothesis 1Sub. 40

Supplementary Figure 10. Results from the modelling based on hypothesis 1Ld. 43

Supplementary References 44

Supplementary Tables

Supplementary Table 1. Description of the specimens included in the study[a].

Specimens	Chronology	Sex	Country	Population - Site	3D[b]	Inst[c]	f/n[d]
Phylogeny sample							**0.37**
H. habilis							0.50
**KNM-ER1470**	~1.8 Ma[93]	M	Kenya	Koobi Fora	PH	NMK	
**KNM-ER1813**	~1.8 Ma[93]	F	Kenya	Koobi Fora	PH	NMK	
H. ergaster							0.33
**KNM-ER3733**	~1.6 Ma[93]	F	Kenya	Koobi Fora	PH	NMK	
**KNM-ER3883**	~1.6 Ma[93]	M	Kenya	Koobi Fora	PH	NMK	
**KNM-WT15000**	1.6-1.4 Ma[94]	M	Kenya	Koobi Fora	PH	NMK	
H. georgicus							0.50
D2282	1.77 Ma[95]	F	Georgia	Dmanisi	PH	IPH	
D2700	1.77 Ma[95]	M	Georgia	Dmanisi	PH	IPH	
Early Neandertal							0.50
**Saccopastore1**	130-250 ka[96]	F	Italy	Saccopatore	PH	US	
**Ehringsdorf H**	~200 ka[19]	M	Germany	Ehringsdorf	PH	IPH	
Near East Neandertal							0
Amud1	50-60 ka[19]	M	Israel	Amud	OP	DC	
Shanidar1	45 ka[97]	M	Irak	Shanidar	PH	MH	
South Europe Neandertal							0.50
**Guattari**	52±12 ka[19]	M	Italy	Monte Circeo	CT	MP	
**Gibraltar1**	45-70 ka[98]	F	UK (Gibraltar)	Gibraltar	CT	NHM	
West Europe Neandertal							0
**LaFerrassie1**	53-66 ka[99]	M	France	La Ferrassie	CT	MH	
**LaChapelle-aux-Saints**	~50 ka[19]	M	France	La Chapelle-aux-Saints	CT	MH	
Early *H. sapiens*							0.33
**Qafzeh6**	100-130 ka[19]	M	Israel	Qafzeh	PH	IPH	
Qafzeh9	100-130 ka[19]	F	Israel	Qafzeh	OP	DC	
**SkhūlV**	88-117 ka[57]	M	Israel	Skhūl	CT	PM	
San							0.60
**Kh-149**	19-20th c.	M	Republic of South Africa	San	CT	DC	
**Kh-1738**	19-20th c.	F	Republic of South Africa	San	CT	DC	
**Kh-1751**	19-20th c.	M	Republic of South Africa	San	CT	DC	
**Kh-3731**	19-20th c.	M	Republic of South Africa	San	CT	DC	
**Kh-3732**	19-20th c.	F	Republic of South Africa	San	CT	DC	
**Kh-3733**	19-20th c.	F	Republic of South Africa	San	PH	DC	
**Kh-5051**	19-20th c.	F	Republic of South Africa	San	CT	DC	
**Kh-5563**	19-20th c.	F	Republic of South Africa	San	PH	MH	
**Kh-6109**	19-20th c.	F	Republic of South Africa	San	CT	DC	
**Kh-AF.30.0.1**	19-20th c.	M	Republic of South Africa	San	CT	DC	
Khoikhoi							0.40
**Kh-1549**	19-20th c.	F	Republic of South Africa	Khoikhoi	PH	MH	
**Kh-1739**	19-20th c.	M	Republic of South Africa	Khoikhoi	CT	DC	
**Kh-1742**	19-20th c.	F	Republic of South Africa	Khoikhoi	CT	DC	
**Kh-1743**	19-20th c.	M	Republic of South Africa	Khoikhoi	CT	DC	
**Kh-1744**	19-20th c.	M	Republic of South Africa	Khoikhoi	CT	DC	
**Kh-1747**	19-20th c.	M	Republic of South Africa	Khoikhoi	CT	DC	
**Kh-3597**	19-20th c.	M	Republic of South Africa	Khoikhoi	PH	MH	
**Kh-3598**	19-20th c.	M	Republic of South Africa	Khoikhoi	PH	MH	
**Kh-3599**	19-20th c.	F	Republic of South Africa	Khoikhoi	PH	MH	
**Kh-5102**	19-20th c.	F	Republic of South Africa	Khoikhoi	PH	DC	
Pygmy - Bayaka							0.50
**Py-9880**	19-20th c.	F	Central African Republic	Bayaka	PH	MH	
**Py-17761**	19-20th c.	F	Central African Republic	Bayaka	PH	MH	
**Py-17766**	19-20th c.	M	Central African Republic	Bayaka	PH	MH	
**Py-17767**	19-20th c.	M	Central African Republic	Bayaka	PH	MH	

**Py-17768**	19-20th c.	M	Central African Republic	Bayaka	PH	MH	
**Py-17769**	19-20th c.	M	Central African Republic	Bayaka	PH	MH	
**Py-17980**	19-20th c.	M	Central African Republic	Bayaka	PH	MH	
**Py-18446**	19-20th c.	F	Central African Republic	Bayaka	PH	MH	
**Py-18449**	19-20th c.	F	Central African Republic	Bayaka	PH	MH	
**Py-23641**	19-20th c.	F	Central African Republic	Bayaka	PH	MH	
Pygmy - Mbuti							0
**Py-5057**	19-20th c.	M	Congo	Mbuti	PH	DC	
**Py-5058**	19-20th c.	M	Congo	Mbuti	CT	DC	
Central Africa							0.21
**AfC-1730**	19-20th c.	M	Congo	Kongo	CT	DC	
**AfC-1774**	19-20th c.	M	Central Africa	Adamawa-Ubangi	CT	DC	
**AfC-1777**	19-20th c.	M	Congo	Kongo	CT	DC	
**AfC-5060**	19-20th c.	M	Congo	Kongo	CT	DC	
**AfC-5424**	19-20th c.	M	Nigeria	Benue	CT	DC	
**AfC-5428**	19-20th c.	M	Nigeria	Benue	CT	DC	
**AfC-5651**	19-20th c.	M	Nigeria	Benue	CT	DC	
**AfC-5701**	19-20th c.	F	Nigeria	Benue	CT	DC	
**AfC-6096**	19-20th c.	M	Central Africa	Adamawa-Ubangi	CT	DC	
**AfC-6097**	19-20th c.	M	Central Africa	Adamawa-Ubangi	CT	DC	
**AfC-AF.20.0.1**	19-20th c.	M	Central Africa	Adamawa-Ubangi	CT	DC	
**AfC-AF.44.0.2**	19-20th c.	M	Nigeria	Yoruba	CT	DC	
**AfC-AF.44.0.4**	19-20th c.	F	Nigeria	Yoruba	CT	DC	
**AfC-AF1257**	19-20th c.	F	Central Africa	Adamawa-Ubangi	CT	DC	
East Africa							0.43
**AF.23.0.109**	19-20th c.	M	Tanzania	Haya	CT	DC	
**AF.23.0.110**	19-20th c.	M	Tanzania	Haya	CT	DC	
**AF.23.0.111**	19-20th c.	M	Tanzania	Haya	CT	DC	
**AF.23.0.112**	19-20th c.	F	Tanzania	Haya	CT	DC	
**AF.23.0.113**	19-20th c.	M	Tanzania	Haya	CT	DC	
**AF.23.0.16**	19-20th c.	M	Tanzania	Haya	CT	DC	
**AF.23.0.18**	19-20th c.	M	Tanzania	Haya	CT	DC	
**AF.23.0.19**	19-20th c.	M	Tanzania	Haya	CT	DC	
**AF.23.0.20**	19-20th c.	F	Tanzania	Haya	CT	DC	
**AF.23.0.21**	19-20th c.	M	Tanzania	Haya	CT	DC	
**AF.23.0.23**	19-20th c.	F	Tanzania	Haya	CT	DC	
**AF.23.0.34**	19-20th c.	F	Tanzania	Haya	CT	DC	
**AF.23.0.42**	19-20th c.	F	Tanzania	Haya	CT	DC	
**AF.23.0.44**	19-20th c.	F	Tanzania	Haya	CT	DC	
West Africa							0.33
**AfW-1725**	19-20th c.	M	Ghana	Akan-Fante	CT	DC	
**AfW-1728**	19-20th c.	M	Guinea	Susu	CT	DC	
**AfW-1729**	19-20th c.	M	Guinea	Susu	CT	DC	
**AfW-1778**	19-20th c.	F	Mali	Gur	CT	DC	
**AfW-5419**	19-20th c.	F	Mali	Mandenka	CT	DC	
**AfW-5423**	19-20th c.	M	Mali	Mandenka	CT	DC	
**AfW-5643**	19-20th c.	M	Ghana	Akan-Fante	CT	DC	
**AfW-6087**	19-20th c.	M	Mali	Mandenka	CT	DC	
**AfW-6089**	19-20th c.	F	Mali	Mandenka	CT	DC	
South Africa							0
**AfS-1732**	19-20th c.	M	Republic of South Africa	Nguni	CT	DC	
**AfS-1733**	19-20th c.	M	Republic of South Africa	Nguni	CT	DC	
**AfS-1734**	19-20th c.	M	Republic of South Africa	Nguni	CT	DC	
**AfS-1735**	19-20th c.	M	Republic of South Africa	Sotho-Tswana	CT	DC	
**AfS-1737**	19-20th c.	M	Republic of South Africa	Xhosa	CT	DC	
**AfS-AF.35.0.1**	19-20th c.	M	Republic of South Africa	Xhosa	CT	DC	
Nilotic							0

**Nil-6092**	19-20th c.	M	Uganda	Teso	CT	DC	
**Nil-6093**	19-20th c.	M	Uganda	Teso	CT	DC	
**Nil-6094**	19-20th c.	M	Uganda	Teso	CT	DC	
**Nil-Af.21.0.1**	19-20th c.	M	Kenya	Turkana	PH	DC	
**Nil-Af.21.0.2**	19-20th c.	M	Kenya	Turkana	PH	DC	
**Nil-Af.21.0.4**	19-20th c.	M	Kenya	Turkana	PH	DC	
**Nil-Af.21.0.5**	19-20th c.	M	Kenya	Turkana	PH	DC	
**Nil-Af.21.0.6**	19-20th c.	M	Kenya	Turkana	PH	DC	
Papua							0.50
**MEL076**	19-20th c.	F	Papua New Guinea	Papuan	PH	DC	
**MEL084**	19-20th c.	M	Papua New Guinea	Papuan	PH	DC	
**MEL085**	19-20th c.	M	Papua New Guinea	Papuan	PH	DC	
**MEL104**	19-20th c.	F	Papua New Guinea	Papuan	CT	DC	
**MEL130**	19-20th c.	F	Papua New Guinea	Papuan	PH	DC	
**MEL153**	19-20th c.	F	Papua New Guinea	Papuan	CT	DC	
**MEL154**	19-20th c.	F	Papua New Guinea	Papuan	CT	DC	
**MEL185**	19-20th c.	M	Papua New Guinea	Papuan	PH	DC	
**MEL197**	19-20th c.	M	Papua New Guinea	Papuan	CT	DC	
**MEL207**	19-20th c.	F	Papua New Guinea	Papuan	PH	DC	
**MEL210**	19-20th c.	F	Papua New Guinea	Papuan	PH	DC	
**MEL214**	19-20th c.	M	Papua New Guinea	Papuan	PH	DC	
**MEL217**	19-20th c.	M	Papua New Guinea	Papuan	PH	DC	
**MEL273**	19-20th c.	M	Papua New Guinea	Papuan	PH	DC	
Australia							0.43
**AUS001**	19-20th c.	M	Australia	Australian	CT	DC	
**AUS028**	19-20th c.	M	Australia	Australian	CT	DC	
**AUS046**	19-20th c.	F	Australia	Australian	CT	DC	
**AUS047**	19-20th c.	F	Australia	Australian	CT	DC	
**AUS059**	19-20th c.	F	Australia	Australian	CT	DC	
**AUS106**	19-20th c.	M	Australia	Australian	CT	DC	
**AUS108**	19-20th c.	M	Australia	Australian	CT	DC	
**AUS122**	19-20th c.	M	Australia	Australian	CT	DC	
**AUS124**	19-20th c.	F	Australia	Australian	CT	DC	
**AUS125**	19-20th c.	F	Australia	Australian	CT	DC	
**AUS126**	19-20th c.	M	Australia	Australian	CT	DC	
**AUS127**	19-20th c.	M	Australia	Australian	CT	DC	
**AUS128**	19-20th c.	M	Australia	Australian	CT	DC	
**AUS131**	19-20th c.	F	Australia	Australian	CT	DC	
North Africa							0.40
**AF.11-NU761**	19-20th c.	M	Soudan (Nubia)	Nubian	CT	DC	
**AF.11.5.033**	5.4-5.1 ka[100]	M	Egypt	Badari	OP	DC	
**AF.11.5.037**	5.4-5.1 ka[100]	M	Egypt	Badari	OP	DC	
**AF.11.5.039**	5.4-5.1 ka[100]	M	Egypt	Badari	OP	DC	
**AF.11.5.040**	5.4-5.1 ka[100]	M	Egypt	Badari	OP	DC	
**AF.11.5.046**	5.4-5.1 ka[100]	F	Egypt	Badari	OP	DC	
**AF.11.5.053**	5.4-5.1 ka[100]	M	Egypt	Badari	OP	DC	
**AF.11.5.054**	5.4-5.1 ka[100]	F	Egypt	Badari	OP	DC	
**AF.11.5.104**	6.3-4.9 ka[100]	M	Egypt	Naqada	OP	DC	
**AF.11.5.116**	6.3-4.9 ka[100]	F	Egypt	Naqada	OP	DC	
**AF.11.5.181**	6.3-4.9 ka[100]	F	Egypt	Naqada	OP	DC	
**AF.11.5.238**	6.3-4.9 ka[100]	M	Egypt	Naqada	CT	DC	
**AF.11.5.417**	6.3-4.9 ka[100]	F	Egypt	Naqada	OP	DC	
**AF.11.5.476**	6.3-4.9 ka[100]	F	Egypt	Naqada	OP	DC	
**AfN-1769**	19-20th c.	M	Egypt	Arab	CT	DC	
South Europe							0.33
**Eu-1036**	19-20th c.	F	France	French	CT	DC	
**Eu-1042**	19-20th c.	M	France	French	CT	DC	
**Eu-1051**	19-20th c.	M	France	French	CT	DC	

**Eu-1065**	19-20th c.	F	France	French	CT	DC	
**Eu-1067**	19-20th c.	M	France	French	CT	DC	
**Eu-1114**	19-20th c.	M	Italy	Italian	CT	DC	
**Eu-1120**	19-20th c.	M	Italy	Italian	CT	DC	
**Eu-2235**	19-20th c.	M	France	Italian	CT	DC	
**Eu.24.00.1**	19-20th c.	M	France	French	CT	DC	
**Eu.42.00.1**	19-20th c.	F	Italy	Italian	CT	DC	
**Eu.42.00.2**	19-20th c.	M	Italy	Italian	CT	DC	
**Eu.42.00.5**	19-20th c.	M	Italy	Italian	CT	DC	
**Eu.43.00.3**	19-20th c.	M	Malta	Maltese	CT	DC	
**Eu.43.00.4**	19-20th c.	F	Malta	Maltese	CT	DC	
**Eu.45.4.1**	3.0-2.2 ka	F	Spain	Minorca	CT	DC	
North Europe							0.43
**Eu-1143**	19-20th c.	M	Austria	Austrian	CT	DC	
**Eu-1150**	19-20th c.	F	Austria	Austrian	CT	DC	
**Eu-1155**	19-20th c.	M	Czech Republic	Czech	CT	DC	
**Eu-1173**	19-20th c.	F	Sweden	Sami	CT	DC	
**Eu-1178**	19-20th c.	M	Denmark	Dane	CT	DC	
**Eu-1181**	19-20th c.	M	Austria	Austrian	CT	DC	
**Eu.1.5.67**	19-20th c.	M	England	English	CT	DC	
**Eu.24.00.2**	19-20th c.	F	Germany	German	CT	DC	
**Eu.26.00.1**	19-20th c.	F	Germany	German	CT	DC	
**Eu.26.00.2**	19-20th c.	M	Germany	German	CT	DC	
**Eu.31.0.1**	19-20th c.	M	Ukraine	Ukrainian	CT	DC	
**Eu.31.00.1**	19-20th c.	M	Russia	Russian	CT	DC	
**Eu.31.00.2**	19-20th c.	F	Russia	Russian	CT	DC	
**Eu.34.4.1**	19-20th c.	F	Hungary	Hungarian	CT	DC	
South Asia							0.21
**SAS-03**	19-20th c.	M	India	Vedda	CT	DC	
**SAS-04**	19-20th c.	M	India	Vedda	CT	DC	
**SAS-05**	19-20th c.	M	India	Vedda	CT	DC	
**SAS-06**	19-20th c.	M	India	Vedda	CT	DC	
**SL3315**	19-20th c.	F	India	Sinhalese	CT	DC	
**SL3317**	19-20th c.	M	India	Sinhalese	CT	DC	
**SL3318**	19-20th c.	M	India	Sinhalese	CT	DC	
**SAS-08**	19-20th c.	F	India	Tamil	CT	DC	
**SAS-11**	19-20th c.	M	India	Tamil	CT	DC	
**SAS-13**	19-20th c.	F	Pakistan	Nagyr	CT	DC	
**SAS-14**	19-20th c.	M	Pakistan	Nagyr	CT	DC	
**SAS-15**	19-20th c.	M	Pakistan	Pashtun	CT	DC	
**SAS-16**	19-20th c.	M	Pakistan	Pashtun	CT	DC	
**SAS-17**	19-20th c.	M	Pakistan	Pashtun	CT	DC	
South East Asia							0.36
**BU02**	19-20th c.	F	Myanmar	Bamar	CT	DC	
**BU04**	19-20th c.	M	Myanmar	Bamar	CT	DC	
**BU08**	19-20th c.	M	Myanmar	Bamar	CT	DC	
**BU10**	19-20th c.	F	Myanmar	Bamar	CT	DC	
**BU14**	19-20th c.	M	Myanmar	Bamar	CT	DC	
**BU16**	19-20th c.	M	Myanmar	Bamar	CT	DC	
**BU19**	19-20th c.	M	Myanmar	Bamar	CT	DC	
**BU21**	19-20th c.	M	Myanmar	Bamar	CT	DC	
**BU24**	19-20th c.	M	Myanmar	Bamar	CT	DC	
**BU26**	19-20th c.	F	Myanmar	Bamar	CT	DC	
**BU28**	19-20th c.	F	Myanmar	Bamar	CT	DC	
**BU29**	19-20th c.	M	Myanmar	Bamar	CT	DC	
**BU31**	19-20th c.	M	Myanmar	Bamar	CT	DC	
**BU32**	19-20th c.	F	Myanmar	Bamar	CT	DC	

East Asia - Japan							0.30
**EAS-1764**	19-20th c.	M	Japan	Ainu	CT	DC	
**EAS24-6**	19-20th c.	M	Japan	Ainu	CT	DC	
**EAS26-2377**	19-20th c.	M	Japan	Japanese	CT	DC	
**EAS28-5430**	19-20th c.	F	Japan	Japanese	PH	DC	
**EAS-4194**	19-20th c.	M	Japan	Japanese	PH	DC	
**EAS-9069**	19-20th c.	M	Japan	Japanese	PH	MH	
**EAS-9071**	19-20th c.	M	Japan	Japanese	PH	MH	
**EAS-9072**	19-20th c.	F	Japan	Japanese	PH	MH	
**EAS-9074**	19-20th c.	M	Japan	Japanese	PH	MH	
**EAS-ORSA0668**	19-20th c.	F	Japan	Japanese	CT	OR	
East Asia - China							0.20
**EAS-1760**	19-20th c.	M	China	Chinese	CT	DC	
**EAS-AS.21.0.7**	19-20th c.	M	China	Chinese	CT	DC	
**EAS-ORSA0426**	19-20th c.	M	China	Chinese	CT	OR	
**EAS-ORSA0427**	19-20th c.	M	China	Chinese	CT	OR	
**EAS-ORSA0550**	19-20th c.	F	China	Chinese	CT	OR	
**EAS-ORSA056**	19-20th c.	M	China	Chinese	CT	OR	
**EAS-ORSA0669**	19-20th c.	M	China	Chinese	CT	OR	
**EAS-ORSA0670**	19-20th c.	F	China	Chinese	CT	OR	
**EAS-ORSA1858**	19-20th c.	M	China	Chinese	CT	OR	
**EAS-ORSA1859**	19-20th c.	M	China	Chinese	CT	OR	
Inuit - Greenland							0.50
**NA123**	19-20th c.	M	Denmark (Greenland)	Inuit	CT	DC	
**NA124**	19-20th c.	F	Denmark (Greenland)	Inuit	CT	DC	
**NA133**	19-20th c.	F	Denmark (Greenland)	Inuit	CT	DC	
**NA134**	19-20th c.	M	Denmark (Greenland)	Inuit	CT	DC	
**NA136**	19-20th c.	M	Denmark (Greenland)	Inuit	CT	DC	
**NA137**	19-20th c.	M	Denmark (Greenland)	Inuit	CT	DC	
**NA138**	19-20th c.	M	Denmark (Greenland)	Inuit	CT	DC	
**NA140**	19-20th c.	M	Denmark (Greenland)	Inuit	CT	DC	
**NA144**	19-20th c.	F	Denmark (Greenland)	Inuit	CT	DC	
**NA145**	19-20th c.	F	Denmark (Greenland)	Inuit	CT	DC	
**NA147**	19-20th c.	M	Denmark (Greenland)	Inuit	CT	DC	
**NA149**	19-20th c.	M	Denmark (Greenland)	Inuit	CT	DC	
**NA150**	19-20th c.	F	Denmark (Greenland)	Inuit	CT	DC	
**NA151**	19-20th c.	F	Denmark (Greenland)	Inuit	CT	DC	
**NA173**	19-20th c.	F	Denmark (Greenland)	Inuit	CT	DC	
Inuit - Alaska							0.62
**99.1-102**	19-20th c.	F	USA (Alaska)	Ipiutak	CT	AM	
**99.1-103**	19-20th c.	M	USA (Alaska)	Ipiutak	CT	AM	
**99.1-161**	19-20th c.	F	USA (Alaska)	Ipiutak	CT	AM	
**99.1-168**	19-20th c.	F	USA (Alaska)	Ipiutak	CT	AM	
**99.1-191**	19-20th c.	F	USA (Alaska)	Ipiutak	CT	AM	
**99.1-196**	19-20th c.	M	USA (Alaska)	Ipiutak	CT	AM	
**99.1-197**	19-20th c.	F	USA (Alaska)	Ipiutak	CT	AM	
**99.1-199**	19-20th c.	F	USA (Alaska)	Ipiutak	CT	AM	
**99.1-204**	19-20th c.	F	USA (Alaska)	Ipiutak	CT	AM	
**99.1-252**	19-20th c.	M	USA (Alaska)	Ipiutak	CT	AM	
**99.1-684**	19-20th c.	F	USA (Alaska)	Ipiutak	CT	AM	
**99.1-87**	19-20th c.	M	USA (Alaska)	Ipiutak	CT	AM	
**99.1-92**	19-20th c.	M	USA (Alaska)	Ipiutak	CT	AM	
North America (native)							0.43
**NA12**	19-20th c.	F	USA (New Mexico)	Zuni	CT	DC	
**NA15**	19-20th c.	F	USA (New Mexico)	Zuni	CT	DC	
**NA23**	19-20th c.	F	USA (New Mexico)	Zuni	CT	DC	
**NA46**	19-20th c.	F	USA (New Mexico)	Zuni	CT	DC	

**NA61**	19-20th c.	M	USA (New Mexico)	Zuni	CT	DC	
**NA68**	19-20th c.	F	USA (Midwest)	Sioux	CT	DC	
**NA72**	19-20th c.	M	USA (Midwest)	Sioux	CT	DC	
**NA74**	19-20th c.	M	Canada (Ontario)	Iroquois	CT	DC	
**NA81**	19-20th c.	M	Canada (Ontario)	Huron	CT	DC	
**NA82**	19-20th c.	M	Canada (Ontario)	Huron	CT	DC	
**NA83**	19-20th c.	M	Canada (Ontario)	Huron	CT	DC	
**NA92**	19-20th c.	M	Canada (British Columbia)	Squamish	CT	DC	
**NA95**	19-20th c.	M	Canada (British Columbia)	Squamish	CT	DC	
**NA97**	19-20th c.	F	Canada (British Columbia)	Squamish	CT	DC	
LMP sample							0
Irhoud 1	~315±34 ka[20]	M	Morroco	Jebel Irhoud	OP	DC	
Omo II	195±95 ka[23]	M	Ethiopia	Omo Valley	PH	NMK	
**LH 18**	200-300 ka[29]	?	Tanzania	Laetoli	CT	TAZ	
**Florisbad**	259±35 ka[31]	?	Republic of South Africa	Florisbad	PH	NM	
**KNM-ES11693**	270-300 ka[28]	?	Kenya	Eliye Springs	PH	NMK	

[a] Bold types indicate when original specimens were examined.

[b] Indicates the scanning method: CT scanner, optical scanner (OP) or photogrammetry (PH)

[c] Indicates where the specimens are curated (DC = Duckworth Laboratory, Cambridge; IPH = Institut de Paléontologie Humaine, Paris; MH = Musée de l'Homme, Paris; SU = Museo di Antropologia, Sapienza Università di Roma; MP = Museo Pigorini, Rome; NHM = Natural History Museum, London; NMK = National Museums of Kenya, Nairobi; NM = National Museum, Bloemfontein; OR = ORSA database, Penn Museum; PM = Peabody Museum, Cambridge; TAZ = National Museum of Tanzania, Dar Es Salaam; AM = American Museum of Natural History, New York, CT scans from[105]); Ma = millions of years; ka = thousands of years.

[d] Ratio f/n indicates the ratio between the number of female versus the number of specimens for each group and for the total phylogeny sample.

Supplementary Table 2. Description of the phylogenetic hypotheses, showing the topology of the trees along with associated split times and references.

Taxa names	Hypothesis 1					Hypothesis 2				
			Branch length	Split times at nodes				Branch length	Split times at nodes	
	Tips/Nodes	To node	(ka)	Nodes	Split (ka)	Tips/Nodes	To node	(ka)	Nodes	Split (ka)
Homo habilis	**1**	-	1800[93]	**30**	**2000**	**1**	-	1800[93]	**30**	**2000**
Homo ergaster	**2**	-	1600[93]	**31**	**1900**	**2**	-	1600[93]	**31**	**1900**
Homo georgicus	**3**	-	1770[95]	**32**	**1800**	**3**	-	1770[95]	**32**	**1800**
**Early Neandertal**	**4**	-	190[19,96]	**33**	**600**	**4**	-	190[19,96]	**33**	**600**
**Near East Neandertal**	**5**	-	55[19,97]	**34**	**300**	**5**	-	55[19,97]	**34**	**300**
**South Europe Neandertal**	**6**	-	55[19,98]	**35**	**150**	**6**	-	55[19,98]	**35**	**150**
**West Europe Neandertal**	**7**	-	59.5[19,99]	**36**	**100**	**7**	-	59.5[19,99]	**36**	**100**
**Early *Homo sapiens***	**8**	-	109[19,57]	**37**	**305**	**8**	-	109[19,57]	**37**	**305**
**San**	**9**	-	0	**38**	**173**	**9**	-	0	**38**	**173**
**Khoikhoi**	**10**	-	0	**39**	**26**	**10**	-	0	**39**	**26**
**Pygmy Bayaka**	**11**	-	0	**40**	**171**	**11**	-	0	**40**	**171**
**Pygmy Mbuti**	**12**	-	0	**41**	**44**	**12**	-	0	**41**	**44**
**Central Africa**	**13**	-	0	**42**	**123**	**13**	-	0	**42**	**123**
**East Africa**	**14**	-	0	**43**	**25**	**14**	-	0	**43**	**25**
**West Africa**	**15**	-	0	**44**	**10**	**15**	-	0	**44**	**10**
**South Africa**	**16**	-	0	**45**	**5**	**16**	-	0	**45**	**5**
**Nilotic**	**17**	-	0	**46**	**4**	**17**	-	0	**46**	**4**
**North Africa**	**18**	-	0	**47**	**90**	**18**	-	0	**47**	**75**
**South Europe**	**19**	-	0	**48**	**37.5**	**19**	-	0	**48**	**63**
**North Europe**	**20**	-	0	**49**	**75**	**20**	-	0	**49**	**24.5**
**South Asia**	**21**	-	0	**50**	**42**	**21**	-	0	**50**	**28**
**Papua**	**22**	-	0	**51**	**24.5**	**22**	-	0	**51**	**27**
**Australia**	**23**	-	0	**52**	**28**	**23**	-	0	**52**	**25**
**South East Asia**	**24**	-	0	**53**	**22**	**24**	-	0	**53**	**22**
**East Asia_Japan**	**25**	-	0	**54**	**14**	**25**	-	0	**54**	**14**
**East Asia_China**	**26**	-	0	**55**	**12**	**26**	-	0	**55**	**12**
**Inuits_Greenland**	**27**	-	0	**56**	**15**	**27**	-	0	**56**	**15**
**Inuits_Alaska**	**28**	-	0	**57**	**2**	**28**	-	0	**57**	**2**
**North America**	**29**	-	0			**29**	-	0		
	**30**	**31**	100			**30**	**31**	100		

**31**	**1**	100
**31**	**32**	100
**32**	**2**	200
**32**	**3**	30
**30**	**33**	1400[69]
**33**	**34**	300[78]
**34**	**4**	110[19,96]
**34**	**35**	150[78]
**35**	**5**	95[19,97]
**35**	**36**	50[78]
**36**	**6**	45[19,98]
**36**	**7**	40.5[19,99]
**33**	**37**	295[69]
**37**	**8**	196[19,57]
**37**	**38**	132[55]
**38**	**39**	147[55]
**39**	**9**	26[55]
**39**	**10**	26[55]
**38**	**40**	2[55]
**40**	**41**	127[55]
**41**	**11**	44[55]
**41**	**12**	44[55]
**40**	**42**	48[55]
**42**	**43**	98[55]
**43**	**44**	15[55]
**44**	**13**	10[55]
**44**	**45**	5[102]
**45**	**14**	5[102]
**45**	**46**	1[102]
**46**	**15**	4[102]
**46**	**16**	4[102]
**43**	**17**	25[55]
**42**	**47**	33[39]
**47**	**48**	52.5[14]
**48**	**18**	37.5[14]

**31**	**1**	100
**31**	**32**	100
**32**	**2**	200
**32**	**3**	30
**30**	**33**	1400[69]
**33**	**34**	300[78]
**34**	**4**	110[19,96]
**34**	**35**	150[78]
**35**	**5**	95[19,97]
**35**	**36**	50[78]
**36**	**6**	45[19,98]
**36**	**7**	40.5[19,99]
**33**	**37**	295[69]
**37**	**8**	196[19,57]
**37**	**38**	132[55]
**38**	**39**	147[55]
**39**	**9**	26[55]
**39**	**10**	26[55]
**38**	**40**	2[55]
**40**	**41**	127[55]
**41**	**11**	44[55]
**41**	**12**	44[55]
**40**	**42**	48[55]
**42**	**43**	98[55]
**43**	**44**	15[55]
**44**	**13**	10[55]
**44**	**45**	5[102]
**45**	**14**	5[102]
**45**	**46**	1[102]
**46**	**15**	4[102]
**46**	**16**	4[102]
**43**	**17**	25[55]
**42**	**47**	48[39]
**47**	**20**	75[39]
**47**	**48**	12[55]

**48**	**19**	37.5[14]
**47**	**49**	15[39]
**49**	**20**	75[39]
**49**	**50**	33[14]
**50**	**51**	17.5[103]
**51**	**21**	24.5[103]
**51**	**22**	24.5[103]
**50**	**52**	14[55]
**52**	**23**	28[55]
**52**	**53**	6[55]
**53**	**54**	8[55]
**54**	**24**	14[55]
**54**	**55**	2[104]
**55**	**25**	12[104]
**55**	**26**	12[104]
**53**	**56**	7[105]
**56**	**57**	13[105]
**57**	**27**	2[105]
**57**	**28**	2[105]
**56**	**29**	15[105]

**48**	**49**	38.5[103]
**49**	**21**	24.5[103]
**49**	**22**	24.5[103]
**48**	**50**	35[55]
**50**	**23**	28[55]
**50**	**51**	1[55]
**51**	**52**	2[14]
**52**	**18**	25[14]
**52**	**19**	25[14]
**51**	**53**	5[55]
**53**	**54**	8[55]
**54**	**24**	14[55]
**54**	**55**	2[104]
**55**	**25**	12[104]
**55**	**26**	12[104]
**53**	**56**	7[105]
**56**	**57**	13[105]
**57**	**27**	2[105]
**57**	**28**	2[105]
**56**	**29**	15[105]

Supplementary Table 3. Description of the PCs from the aligned tree data for Hypotheses 1 and 2

	Eigenvalues	% Variance	Cumulative %
**PC1**	0.002221	65.61	65.61
**PC2**	0.000447	13.21	78.82
**PC3**	0.000196	5.78	84.59
**PC4**	0.000103	3.04	87.64
**PC5**	8.79E-05	2.60	90.23
**PC6**	6.41E-05	1.89	92.13
**PC7**	4.67E-05	1.38	93.50
**PC8**	4.09E-05	1.21	94.71
**PC9**	3.14E-05	0.93	95.64
**PC10**	2.87E-05	0.85	96.49
**PC11**	2.12E-05	0.63	97.11
**PC12**	1.91E-05	0.56	97.68
**PC13**	1.70E-05	0.50	98.18
**PC14**	1.07E-05	0.32	98.49
**PC15**	9.33E-06	0.28	98.77
**PC16**	7.61E-06	0.22	98.99
**PC17**	7.40E-06	0.22	99.21
**PC18**	6.16E-06	0.18	99.39
**PC19**	3.81E-06	0.11	99.51
**PC20**	3.29E-06	0.10	99.60
**PC21**	2.84E-06	0.08	99.69
**PC22**	2.31E-06	0.07	99.76
**PC23**	2.21E-06	0.07	99.82
**PC24**	1.65E-06	0.05	99.87
**PC25**	1.60E-06	0.05	99.92
**PC26**	1.12E-06	0.03	99.95
**PC27**	8.80E-07	0.03	99.98
**PC28**	7.96E-07	0.02	100.00

PCs scores are used as variables to run the ancestral reconstruction

Supplementary Table 4. Values for the phylogenetic signal K_{mult} (i.e. multivariate K-statistics from Blomberg's K[106]) for hypotheses 1, 2, 1b and 2b.

	PC1 to PC3 (85% of variation)				***PC1 to PC8 (95% of variation)***				***All PCs (100% variation)***				***Landmarks coordinates (100% variation)***	
	**K_{mult}**	**Crit. test stat.**	**Est. power**	***p***	**K_{mult}**	**Crit. test stat.**	**Est. power**	***p***	**K_{mult}**	**Crit. test stat.**	**Est. power**	***p***	**K_{mult}**	***p***
**Hypothesis 1**	1.39	0.0211	1.000	0.001	0.543	0.0190	1.000	0.001	0.386	0.0188	1.000	0.001	0.385	0.001
**Hypothesis 2**	1.18	0.0197	1.000	0.001	0.539	0.0192	1.000	0.001	0.362	0.0175	1.000	0.001	0.368	0.001
**Hypothesis 1b**	1.13	0.0196	1.000	0.001	0.47	0.0171	1.000	0.001	0.334	0.0164	1.000	0.001	0.337	0.001
**Hypothesis 2b**	1.11	0.0217	1.000	0.001	0.48	0.0183	1.000	0.001	0.338	0.0182	1.000	0.001	0.338	0.001

K_{mult} is computed for the three first PCs, the eight first PCs and the 28 PCs[37], as well as for the aligned landmarks coordinates[38]. In all cases, the phylogenetic signals computed present higher values for Hypothesis 1.

Supplementary Table 5. Description of the PCs from the aligned tree data for Hypotheses 1b and 2b

	Eigenvalues	% Variance	Cumulative %
**PC1**	0.0024	66.48	66.48
**PC2**	0.000516	14.28	80.76
**PC3**	0.000186	5.16	85.91
**PC4**	0.000118	3.26	89.17
**PC5**	8.36E-05	2.32	91.48
**PC6**	6.67E-05	1.85	93.33
**PC7**	4.88E-05	1.35	94.69
**PC8**	3.92E-05	1.09	95.77
**PC9**	3.43E-05	0.95	96.72
**PC10**	2.55E-05	0.71	97.43
**PC11**	2.03E-05	0.56	97.99
**PC12**	1.50E-05	0.42	98.40
**PC13**	1.38E-05	0.38	98.79
**PC14**	8.93E-06	0.25	99.03
**PC15**	7.45E-06	0.21	99.24
**PC16**	6.96E-06	0.19	99.43
**PC17**	4.15E-06	0.11	99.55
**PC18**	3.85E-06	0.11	99.65
**PC19**	3.04E-06	0.08	99.74
**PC20**	2.65E-06	0.07	99.81
**PC21**	2.21E-06	0.06	99.87
**PC22**	1.92E-06	0.05	99.93
**PC23**	1.51E-06	0.04	99.97
**PC24**	1.14E-06	0.03	100.00

PCs scores are used as variables to run the ancestral reconstruction

Supplementary Table 6. Maximum, average and standard deviation of the surface deviations between the different vLCAs (mm).

Reference	Test	Max. positive	Max. negative	Avg. positive	Avg. negative	SD
**vLCA 1**	**vLCA 2**	0.16	-0.15	0.05	-0.05	0.06
**vLCA 1**	**vLCA 1b**	1.00	-0.98	0.24	-0.22	0.30
**vLCA 2**	**vLCA 2b**	2.96	-2.45	0.62	-0.58	0.58
**vLCA 1b**	**vLCA 2b**	3.13	-2.84	0.78	-0.74	0.98
**vLCA 1**	**vLCA 1NS**	0.62	-0.50	0.11	-0.10	0.13
**vLCA 1**	**vLCA 1Sub**	2.93	-3.01	0.30	-0.35	0.51
**vLCA 1**	**vLCA 1Ld**	6.65	6.30	1.03	-1.08	1.65

Supplementary Table 7. African LMP specimens' percentage of preserved landmarks for GMM analyses

Ld Configuration	Nb of Ld	% of preserved Ld for the African LMP specimens				
		Irhoud 1	KNM-ES 11693	Florisbad	Omo II	LH 18
**A (Full)**	255	96	66	60	56	55
**B (Calvaria)**	148	97	79	**46**	96	95
**C (Florisbad)**	156	100	62	100	**40**	**40**
**D (ES11693)**	182	98	100	57	63	62

For preserved landmarks < 55% the specimens where not included in the analysis. Irhoud 1 and KNM-ES 11693 were included in the four analyses while Omo II, LH 18 were not included in analysis C and Florisbad in analyses B.

Supplementary Table 8. Description of the between group PCs – Analysis A

	Eigenvalues	% Variance	Cumulative %
**bgPC 1**	0.00032	28.89	28.89
**bgPC 2**	0.000266	24.04	52.93
**bgPC 3**	0.000119	10.74	63.66
**bgPC 4**	8.86E-05	8.00	71.67
**bgPC 5**	5.72E-05	5.17	76.83
**bgPC 6**	5.08E-05	4.59	81.42
**bgPC 7**	3.59E-05	3.24	84.66
**bgPC 8**	2.66E-05	2.41	87.07
**bgPC 9**	2.05E-05	1.85	88.92
**bgPC 10**	1.78E-05	1.61	90.53
**bgPC 11**	1.53E-05	1.38	91.92
**bgPC 12**	1.28E-05	1.15	93.07
**bgPC 13**	1.23E-05	1.11	94.18
**bgPC 14**	9.87E-06	0.89	95.07
**bgPC 15**	7.75E-06	0.70	95.77
**bgPC 16**	7.20E-06	0.65	96.42
**bgPC 17**	6.16E-06	0.56	96.98
**bgPC 18**	5.49E-06	0.50	97.48
**bgPC 19**	5.10E-06	0.46	97.94
**bgPC 20**	4.64E-06	0.42	98.36
**bgPC 21**	3.68E-06	0.33	98.69
**bgPC 22**	3.04E-06	0.27	98.96
**bgPC 23**	2.63E-06	0.24	99.20
**bgPC 24**	2.36E-06	0.21	99.41
**bgPC 25**	1.82E-06	0.16	99.58
**bgPC 26**	1.51E-06	0.14	99.71
**bgPC 27**	1.08E-06	0.10	99.81
**bgPC 28**	9.52E-07	0.09	99.90
**bgPC 29**	7.37E-07	0.07	99.96
**bgPC 30**	3.87E-07	0.03	100.00
**bgPC 31**	1.11E-08	0.00	100.00

Supplementary Table 9. Description of the between group PCs – Analysis B

	Eigenvalues	% Variance	Cumulative %
**bgPC 1**	0.0004178	37.09	37.09
**bgPC 2**	0.0002665	23.65	60.74
**bgPC 3**	0.0001207	10.71	71.46
**bgPC 4**	8.29E-05	7.36	78.82
**bgPC 5**	5.07E-05	4.50	83.31
**bgPC 6**	3.96E-05	3.51	86.83
**bgPC 7**	2.55E-05	2.26	89.09
**bgPC 8**	2.37E-05	2.10	91.19
**bgPC 9**	2.10E-05	1.87	93.05
**bgPC 10**	1.73E-05	1.54	94.59
**bgPC 11**	1.23E-05	1.10	95.69
**bgPC 12**	8.95E-06	0.79	96.48
**bgPC 13**	8.71E-06	0.77	97.26
**bgPC 14**	6.03E-06	0.54	97.79
**bgPC 15**	5.80E-06	0.52	98.31
**bgPC 16**	4.57E-06	0.41	98.71
**bgPC 17**	3.36E-06	0.30	99.01
**bgPC 18**	2.47E-06	0.22	99.23
**bgPC 19**	2.31E-06	0.21	99.43
**bgPC 20**	1.63E-06	0.14	99.58
**bgPC 21**	1.43E-06	0.13	99.71
**bgPC 22**	1.10E-06	0.10	99.80
**bgPC 23**	9.32E-07	0.08	99.89
**bgPC 24**	5.17E-07	0.05	99.93
**bgPC 25**	4.60E-07	0.04	99.97
**bgPC 26**	1.54E-07	0.01	99.99
**bgPC 27**	1.15E-07	0.01	100.00
**bgPC 28**	2.16E-08	0.00	100.00
**bgPC 29**	8.25E-09	0.00	100.00
**bgPC 30**	3.19E-09	0.00	100.00

Supplementary Table 10. Description of the between group PCs – Analysis C

	Eigenvalues	% Variance	Cumulative %
**bgPC 1**	0.0004149	34.56	34.56
**bgPC 2**	0.0003213	26.76	61.32
**bgPC 3**	0.0001306	10.88	72.20
**bgPC 4**	7.59E-05	6.32	78.52
**bgPC 5**	6.53E-05	5.44	83.97
**bgPC 6**	4.36E-05	3.63	87.59
**bgPC 7**	2.77E-05	2.30	89.90
**bgPC 8**	2.18E-05	1.81	91.71
**bgPC 9**	2.11E-05	1.75	93.47
**bgPC 10**	1.37E-05	1.14	94.61
**bgPC 11**	1.22E-05	1.02	95.63
**bgPC 12**	9.28E-06	0.77	96.40
**bgPC 13**	7.49E-06	0.62	97.02
**bgPC 14**	6.79E-06	0.57	97.59
**bgPC 15**	5.61E-06	0.47	98.06
**bgPC 16**	4.33E-06	0.36	98.42
**bgPC 17**	4.24E-06	0.35	98.77
**bgPC 18**	3.47E-06	0.29	99.06
**bgPC 19**	2.60E-06	0.22	99.28
**bgPC 20**	2.46E-06	0.20	99.48
**bgPC 21**	1.75E-06	0.15	99.63
**bgPC 22**	1.60E-06	0.13	99.76
**bgPC 23**	1.02E-06	0.09	99.84
**bgPC 24**	8.27E-07	0.07	99.91
**bgPC 25**	5.44E-07	0.05	99.96
**bgPC 26**	2.99E-07	0.02	99.98
**bgPC 27**	1.34E-07	0.01	99.99
**bgPC 28**	6.48E-08	0.01	100.00
**bgPC 29**	2.62E-09	0.00	100.00

Supplementary Table 11. Description of the between group PCs – Analysis D

	Eigenvalues	% Variance	Cumulative %
**bgPC 1**	0.000329	32.84	32.84
**bgPC 2**	0.0002363	23.59	56.43
**bgPC 3**	0.0001221	12.19	68.62
**bgPC 4**	7.11E-05	7.10	75.72
**bgPC 5**	4.56E-05	4.55	80.27
**bgPC 6**	3.69E-05	3.68	83.95
**bgPC 7**	2.69E-05	2.68	86.63
**bgPC 8**	2.10E-05	2.10	88.73
**bgPC 9**	1.88E-05	1.88	90.61
**bgPC 10**	1.74E-05	1.74	92.35
**bgPC 11**	1.26E-05	1.26	93.61
**bgPC 12**	1.03E-05	1.03	94.63
**bgPC 13**	9.17E-06	0.92	95.55
**bgPC 14**	8.12E-06	0.81	96.36
**bgPC 15**	6.35E-06	0.63	96.99
**bgPC 16**	5.29E-06	0.53	97.52
**bgPC 17**	4.52E-06	0.45	97.97
**bgPC 18**	3.63E-06	0.36	98.33
**bgPC 19**	3.41E-06	0.34	98.67
**bgPC 20**	2.81E-06	0.28	98.96
**bgPC 21**	2.25E-06	0.23	99.18
**bgPC 22**	2.08E-06	0.21	99.39
**bgPC 23**	1.91E-06	0.19	99.58
**bgPC 24**	1.00E-06	0.10	99.68
**bgPC 25**	9.71E-07	0.10	99.78
**bgPC 26**	8.56E-07	0.09	99.86
**bgPC 27**	6.77E-07	0.07	99.93
**bgPC 28**	3.95E-07	0.04	99.97
**bgPC 29**	2.57E-07	0.03	99.99
**bgPC 30**	6.16E-08	0.01	100.00
**bgPC 31**	1.59E-09	0.00	100.00

Supplementary Table 12. Description of the PCs from the aligned tree data for Hypothesis 1 using row data before sliding and data after sliding semilandmarks

	Eigenvalues	% Variance	Cumulative %
**PC1**	0.002276	64.21	64.21
**PC2**	0.000485	13.68	77.89
**PC3**	0.000198	5.58	83.47
**PC4**	0.000107	3.01	86.49
**PC5**	8.56E-05	2.42	88.90
**PC6**	6.45E-05	1.82	90.72
**PC7**	5.35E-05	1.51	92.23
**PC8**	4.15E-05	1.17	93.40
**PC9**	3.98E-05	1.12	94.52
**PC10**	2.78E-05	0.78	95.31
**PC11**	2.52E-05	0.71	96.02
**PC12**	2.15E-05	0.61	96.63
**PC13**	1.81E-05	0.51	97.14
**PC14**	1.41E-05	0.40	97.53
**PC15**	1.18E-05	0.33	97.87
**PC16**	1.05E-05	0.30	98.16
**PC17**	8.54E-06	0.24	98.40
**PC18**	7.27E-06	0.21	98.61
**PC19**	6.41E-06	0.18	98.79
**PC20**	5.66E-06	0.16	98.95
**PC21**	4.94E-06	0.14	99.09
**PC22**	4.12E-06	0.12	99.21
**PC23**	3.17E-06	0.09	99.29
**PC24**	2.84E-06	0.08	99.37
**PC25**	2.65E-06	0.07	99.45
**PC26**	2.53E-06	0.07	99.52
**PC27**	2.12E-06	0.06	99.58
**PC28**	1.99E-06	0.06	99.64
**PC29**	1.54E-06	0.04	99.68
**PC30**	1.32E-06	0.04	99.72
**PC31**	1.26E-06	0.04	99.75
**PC32**	1.05E-06	0.03	99.78
**PC33**	9.61E-07	0.03	99.81
**PC34**	8.99E-07	0.03	99.84
**PC35**	7.91E-07	0.02	99.86
**PC36**	6.80E-07	0.02	99.88
**PC37**	6.49E-07	0.02	99.89
**PC38**	5.51E-07	0.02	99.91
**PC39**	4.62E-07	0.01	99.92
**PC40**	3.98E-07	0.01	99.93
**PC41**	3.39E-07	0.01	99.94
**PC42**	3.27E-07	0.01	99.95
**PC43**	2.61E-07	0.01	99.96
**PC44**	2.29E-07	0.01	99.97
**PC45**	2.21E-07	0.01	99.97
**PC46**	1.68E-07	0.00	99.98
**PC47**	1.40E-07	0.00	99.98
**PC48**	1.26E-07	0.00	99.99
**PC49**	1.02E-07	0.00	99.99
**PC50**	8.92E-08	0.00	99.99
**PC51**	8.02E-08	0.00	99.99
**PC52**	6.31E-08	0.00	100.00

**PC53**	6.00E-08	0.00	100.00
**PC54**	3.69E-08	0.00	100.00
**PC55**	3.10E-08	0.00	100.00
**PC56**	2.29E-08	0.00	100.00
**PC57**	1.51E-08	0.00	100.00

PCs scores are used as variables to run the ancestral reconstruction

Supplementary Table 13. Description of the between group PCs using a sub-sample of 239 landmarks

	Eigenvalues	% Variance	Cumulative %
**bgPC 1**	0.000325895	29.12%	29.12%
**bgPC 2**	0.000274239	24.50%	53.62%
**bgPC 3**	0.000119148	10.65%	64.26%
**bgPC 4**	8.89E-05	7.95%	72.21%
**bgPC 5**	5.78E-05	5.16%	77.37%
**bgPC 6**	4.87E-05	4.35%	81.72%
**bgPC 7**	3.61E-05	3.23%	84.95%
**bgPC 8**	2.54E-05	2.27%	87.22%
**bgPC 9**	2.08E-05	1.86%	89.07%
**bgPC 10**	1.77E-05	1.58%	90.66%
**bgPC 11**	1.49E-05	1.33%	91.99%
**bgPC 12**	1.30E-05	1.16%	93.15%
**bgPC 13**	1.28E-05	1.15%	94.30%
**bgPC 14**	9.29E-06	0.83%	95.13%
**bgPC 15**	7.93E-06	0.71%	95.84%
**bgPC 16**	7.41E-06	0.66%	96.50%
**bgPC 17**	5.91E-06	0.53%	97.03%
**bgPC 18**	5.39E-06	0.48%	97.51%
**bgPC 19**	5.05E-06	0.45%	97.96%
**bgPC 20**	4.27E-06	0.38%	98.34%
**bgPC 21**	3.88E-06	0.35%	98.69%
**bgPC 22**	3.15E-06	0.28%	98.97%
**bgPC 23**	2.51E-06	0.22%	99.19%
**bgPC 24**	2.32E-06	0.21%	99.40%
**bgPC 25**	1.79E-06	0.16%	99.56%
**bgPC 26**	1.56E-06	0.14%	99.70%
**bgPC 27**	1.12E-06	0.10%	99.80%
**bgPC 28**	1.01E-06	0.09%	99.89%
**bgPC 29**	7.73E-07	0.07%	99.96%
**bgPC 30**	4.07E-07	0.04%	100.00%
**bgPC 31**	3.61E-08	0.00%	100.00%
**bgPC 32**	4.68E-09	0.00%	100.00%

Supplementary Table 14. Values for the phylogenetic signal K_{mult} (i.e. multivariate extension of Blomberg's K-statistics[106]) of phylogenetic hypotheses 1, 1NS, 1Sub and 1Ld.

	PC1 to PC3 (85% of variation)				***PC1 to PC8 (95% of variation)***				***All PCs (100% variation)***				***Landmarks coordinates (100% variation)***	
	**K_{mult}**	**Crit. test stat.**	**Est. power**	***p***	**K_{mult}**	**Crit. test stat.**	**Est. power**	***p***	**K_{mult}**	**Crit. test stat.**	**Est. power**	***p***	**K_{mult}**	***p***
**Hypothesis 1**	1.35	0.0211	1.000	0.001	0.549	0.0197	1.000	0.001	0.384	0.0188	1.000	0.001	0.385	0.001
**Hypothesis 1 NS**	1.33	0.0201	1.000	0.001	0.583	0.0193	1.000	0.001	0.352	0.0182	1.000	0.001	0.354	0.001
**Hypothesis 1 Sub**	1.61	0.0209	1.000	0.001	0.662	0.0185	1.000	0.001	0.400	0.0186	1.000	0.001	0.400	0.001
**Hypothesis 1 Ld**	1.10	0.0198	1.000	0.001	0.569	0.0176	1.000	0.001	0.274	0.0169	1.000	0.001	0.278	0.001

K_{mult} is computed for the three first PCs, the eight first PCs and the 28 PCs[37], as well as for the aligned landmarks coordinates[38]. The phylogenetic signals computed are similar for the original hypothesis and the non-slid hypothesis. Reducing the landmarks and semi-landmarks number seems to increase the phylogenetic signal (hypothesis 1 Sub) but the use of only landmarks reduces the value of K_{mult}.

Supplementary Table 15. Description of the between group PCs using a subsample of 53 landmarks

	Eigenvalues	% Variance	Cumulative %
**bgPC 1**	0.0004984	31.30%	31.30%
**bgPC 2**	0.0002857	17.94%	49.24%
**bgPC 3**	0.0001334	8.38%	57.62%
**bgPC 4**	0.0001157	7.27%	64.89%
**bgPC 5**	0.0001064	6.68%	71.57%
**bgPC 6**	8.81E-05	5.53%	77.10%
**bgPC 7**	5.53E-05	3.47%	80.57%
**bgPC 8**	5.44E-05	3.41%	83.99%
**bgPC 9**	3.17E-05	1.99%	85.98%
**bgPC 10**	2.86E-05	1.80%	87.78%
**bgPC 11**	2.80E-05	1.76%	89.53%
**bgPC 12**	2.50E-05	1.57%	91.10%
**bgPC 13**	2.02E-05	1.27%	92.37%
**bgPC 14**	1.80E-05	1.13%	93.51%
**bgPC 15**	1.63E-05	1.02%	94.53%
**bgPC 16**	1.29E-05	0.81%	95.34%
**bgPC 17**	1.25E-05	0.78%	96.12%
**bgPC 18**	1.05E-05	0.66%	96.78%
**bgPC 19**	9.47E-06	0.59%	97.38%
**bgPC 20**	8.68E-06	0.54%	97.92%
**bgPC 21**	6.35E-06	0.40%	98.32%
**bgPC 22**	6.00E-06	0.38%	98.70%
**bgPC 23**	4.66E-06	0.29%	98.99%
**bgPC 24**	3.93E-06	0.25%	99.24%
**bgPC 25**	3.01E-06	0.19%	99.43%
**bgPC 26**	2.82E-06	0.18%	99.60%
**bgPC 27**	2.29E-06	0.14%	99.75%
**bgPC 28**	2.07E-06	0.13%	99.88%
**bgPC 29**	1.31E-06	0.08%	99.96%
**bgPC 30**	5.40E-07	0.03%	99.99%
**bgPC 31**	9.67E-08	0.01%	100.00%
**bgPC 32**	1.19E-08	0.00%	100.00%
**bgPC 33**	8.98E-10	0.00%	100.00%
**bgPC 34**	1.07E-10	0.00%	100.00%

Supplementary Table 16. Description of the PCs from the aligned tree data for Hypothesis 1 using a sub-sample of 239 landmarks and semilandmarks

	Eigenvalues	% Variance	Cumulative %
**PC1**	0.00215	61.75	61.75
**PC2**	0.000431	12.39	74.14
**PC3**	0.000235	6.74	80.88
**PC4**	0.00013	3.74	84.62
**PC5**	0.000113	3.25	87.87
**PC6**	8.54E-05	2.45	90.32
**PC7**	5.51E-05	1.58	91.91
**PC8**	4.87E-05	1.40	93.31
**PC9**	4.50E-05	1.29	94.60
**PC10**	3.26E-05	0.93	95.53
**PC11**	2.69E-05	0.77	96.31
**PC12**	2.51E-05	0.72	97.03
**PC13**	2.09E-05	0.60	97.63
**PC14**	1.67E-05	0.48	98.11
**PC15**	1.43E-05	0.41	98.52
**PC16**	9.14E-06	0.26	98.78
**PC17**	8.22E-06	0.24	99.02
**PC18**	6.76E-06	0.19	99.21
**PC19**	5.05E-06	0.15	99.36
**PC20**	4.50E-06	0.13	99.49
**PC21**	3.87E-06	0.11	99.60
**PC22**	3.18E-06	0.09	99.69
**PC23**	2.56E-06	0.07	99.76
**PC24**	2.29E-06	0.07	99.83
**PC25**	2.14E-06	0.06	99.89
**PC26**	1.48E-06	0.04	99.93
**PC27**	1.22E-06	0.03	99.97
**PC28**	1.18E-06	0.03	100.00

PCs scores are used as variables to run the ancestral reconstruction

Supplementary Table 17. Description of the PCs from the aligned tree data for Hypothesis 1 using a sub-sample of 53 landmarks

	Eigenvalues	% Variance	Cumulative %
**PC1**	0.001969	48.92	48.92
**PC2**	0.0004913	12.21	61.13
**PC3**	0.0004103	10.19	71.33
**PC4**	0.000235	5.84	77.17
**PC5**	0.0001923	4.78	81.95
**PC6**	0.0001393	3.46	85.41
**PC7**	0.0001027	2.55	87.96
**PC8**	8.91E-05	2.21	90.17
**PC9**	8.19E-05	2.04	92.21
**PC10**	5.56E-05	1.38	93.59
**PC11**	5.19E-05	1.29	94.88
**PC12**	4.12E-05	1.02	95.90
**PC13**	3.02E-05	0.75	96.65
**PC14**	2.44E-05	0.61	97.26
**PC15**	1.97E-05	0.49	97.75
**PC16**	1.88E-05	0.47	98.22
**PC17**	1.66E-05	0.41	98.63
**PC18**	9.86E-06	0.24	98.87
**PC19**	8.25E-06	0.20	99.08
**PC20**	7.89E-06	0.20	99.28
**PC21**	6.41E-06	0.16	99.44
**PC22**	5.09E-06	0.13	99.56
**PC23**	4.63E-06	0.12	99.68
**PC24**	3.45E-06	0.09	99.76
**PC25**	3.09E-06	0.08	99.84
**PC26**	2.60E-06	0.06	99.90
**PC27**	1.99E-06	0.05	99.95
**PC28**	1.88E-06	0.05	100.00

PCs scores are used as variables to run the ancestral reconstruction

Supplementary Figures

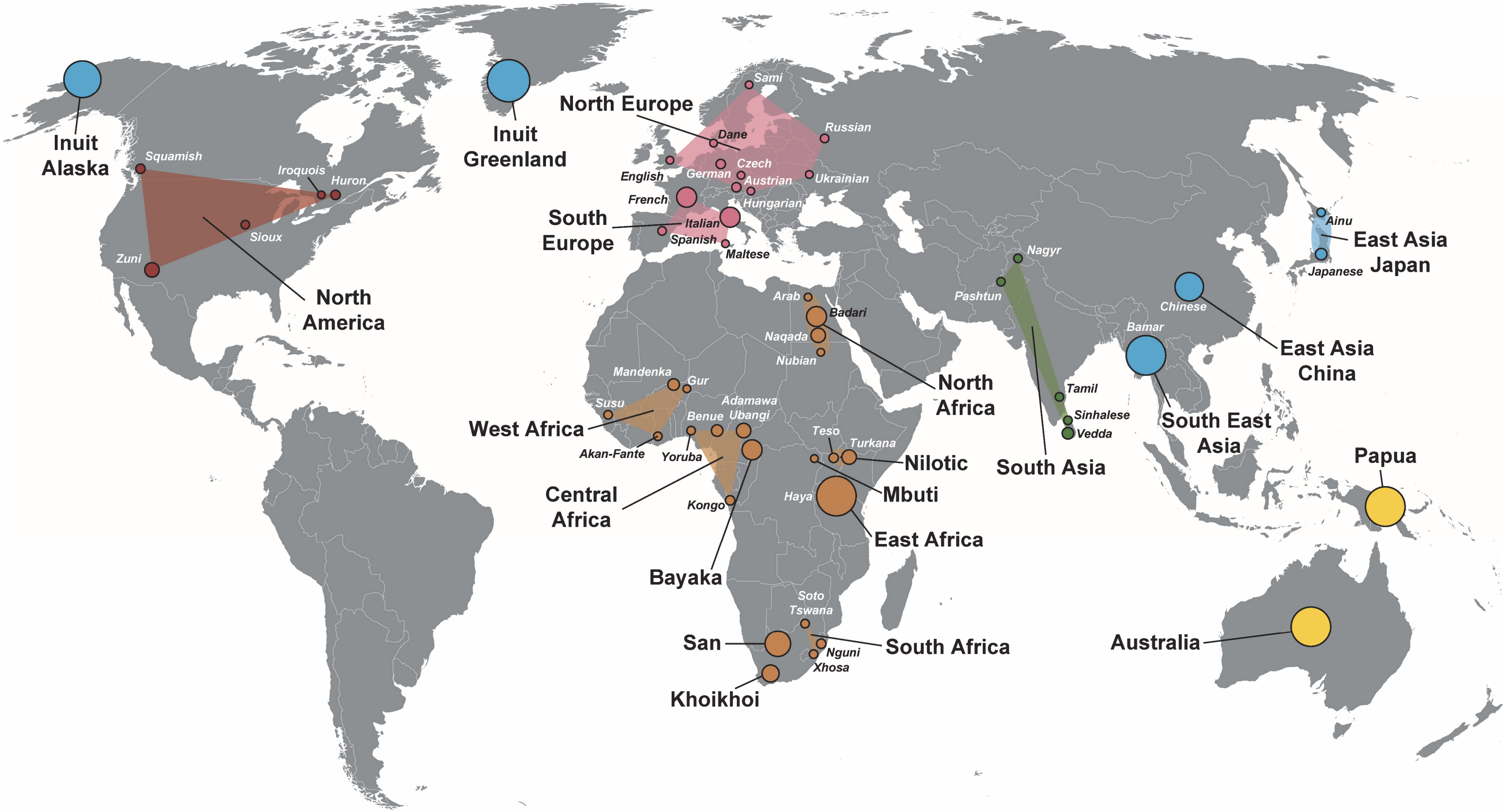

Supplementary Figure 1. World map showing the geographical and ethnical origin of the sampled population.

Circles size is proportional with population size. World map modified from World with Countries - Single Color by FreeVectorMaps.com.

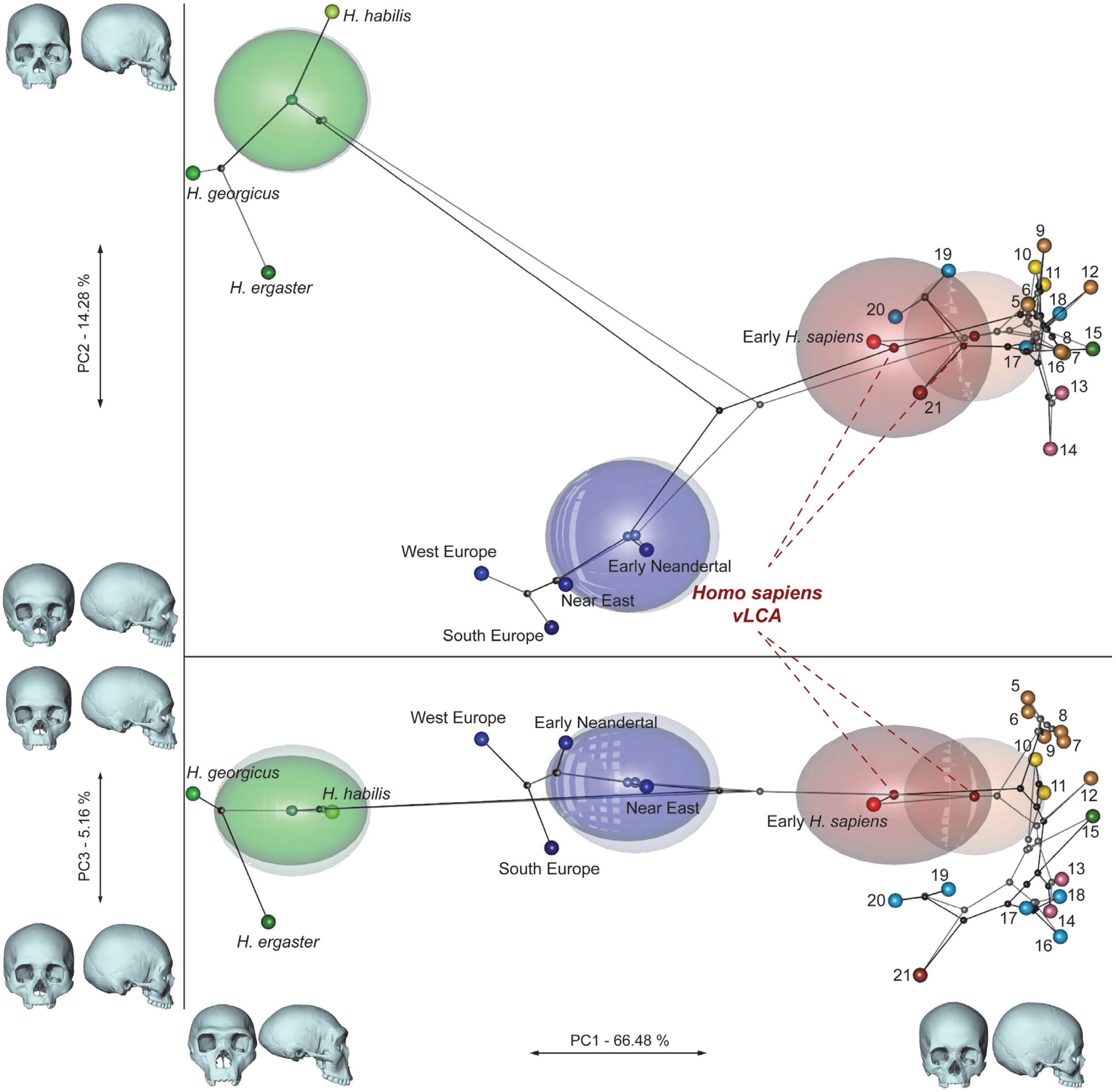

Supplementary Figure 2. Projection of the phylogenies 1b (black) and 2b (grey) in the morphospace.

The associated shape deformations are displayed next to each PC. Each node represents estimated ancestors shapes along with 95% confidence envelopes. Modern human populations as follow: 1 to 9 Sub-Saharan Africa; 10-11 Oceania, 12 North Africa, 13-14 Europe, 15 South Asia, 16 to 20 East Asia, 21 North America (see Fig. 1, Supplementary Fig. 1 and Supplementary Table 1). Source data are provided as a Source Data file.

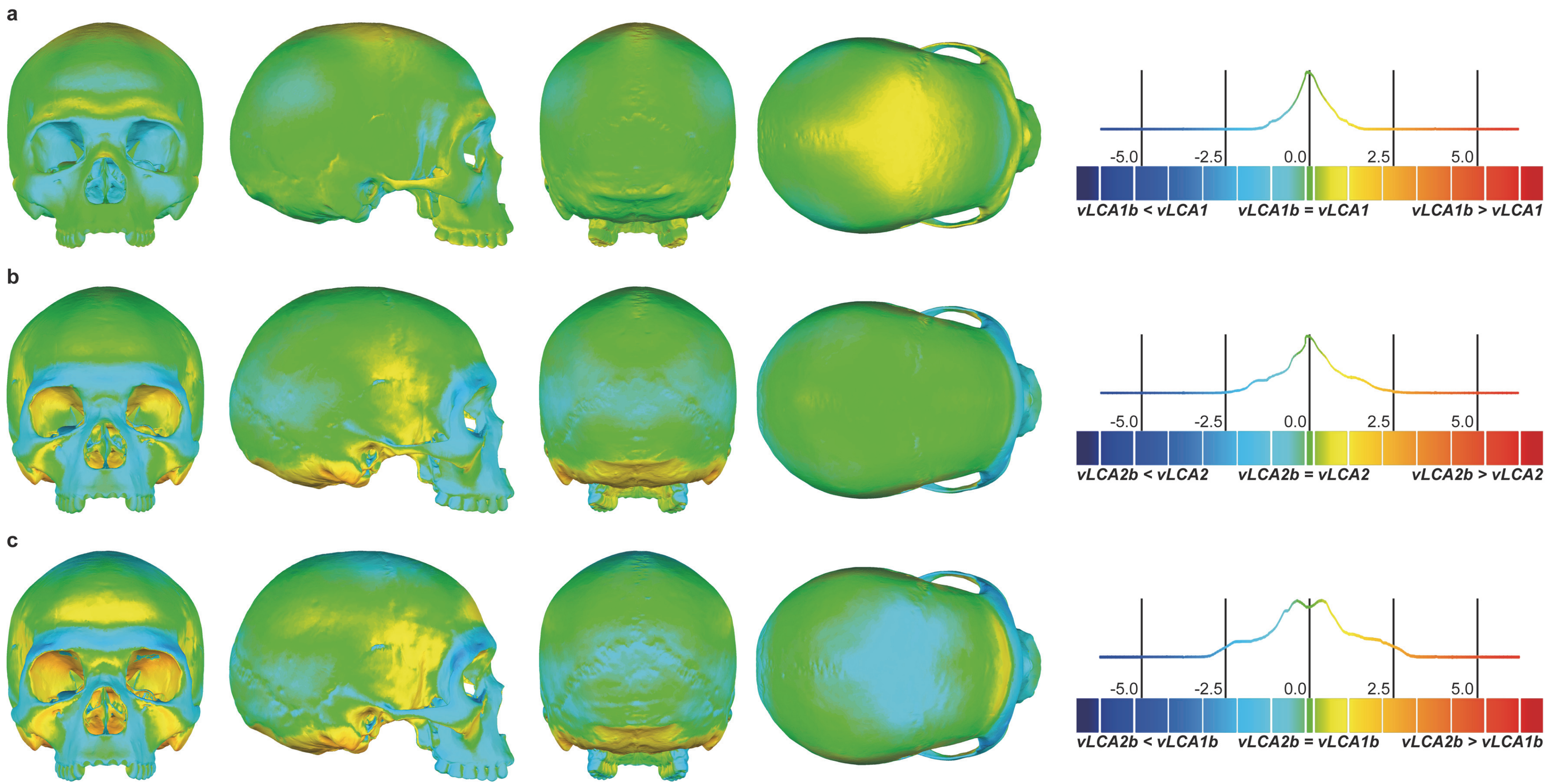

Supplementary Figure 3. Surface deviation analyses comparing the vLCAs' morphologies.

Norma frontalis, *lateralis*, *verticalis*, *occipitalis* views of the result of the surface deviation of vLCA 1 vs 1b (a), vLCA 2 vs 2b, and vLCA 1b vs 2b (c). The histograms indicate the distribution of the deviation in mm between each vertex of the 3d models.

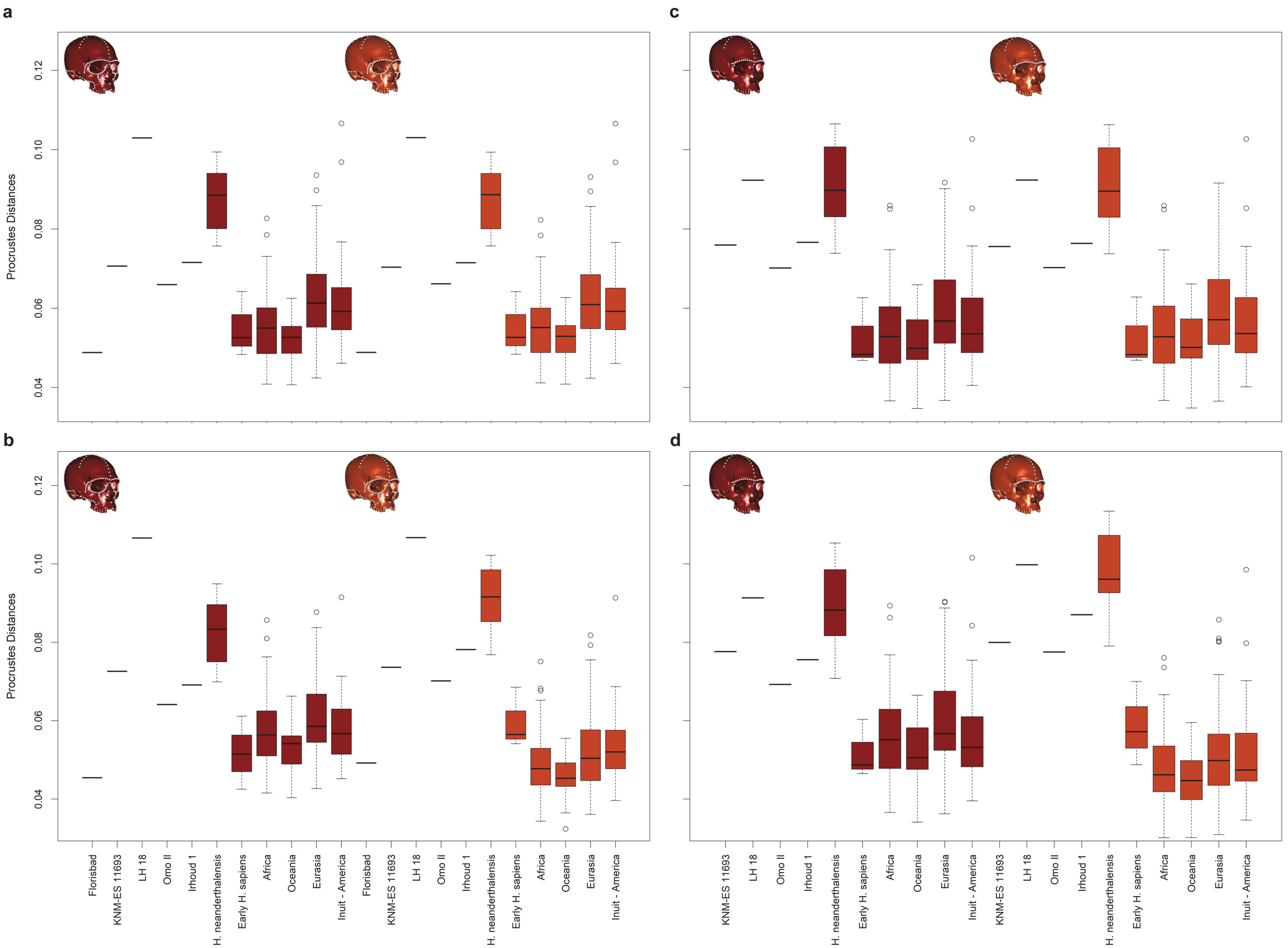

a
b
c
d
Procrustes Distances
0.04
0.06
0.08
0.10
0.12
Florisbad
KNM-ES 11693
LH 18
Omo II
Irhoud 1
H. neanderthalensis
Early H. sapiens
Africa
Oceania
Eurasia
Inuit - America

Supplementary Figure 4. Box-and-whisker plots showing the Procrustes distances between the vLCAs and the comparative sample for analyses A and B.

(a) and (c) show the distances to vLCAs 1 and 2 and (b) and (d) present the distances to vLCAs 1b and 2b. Coloured boxes indicate the interquartile range (25 to 75%) around the median (vertical line); the dotted lines indicate the maximum and minimum range for each population, the circles are outliers.

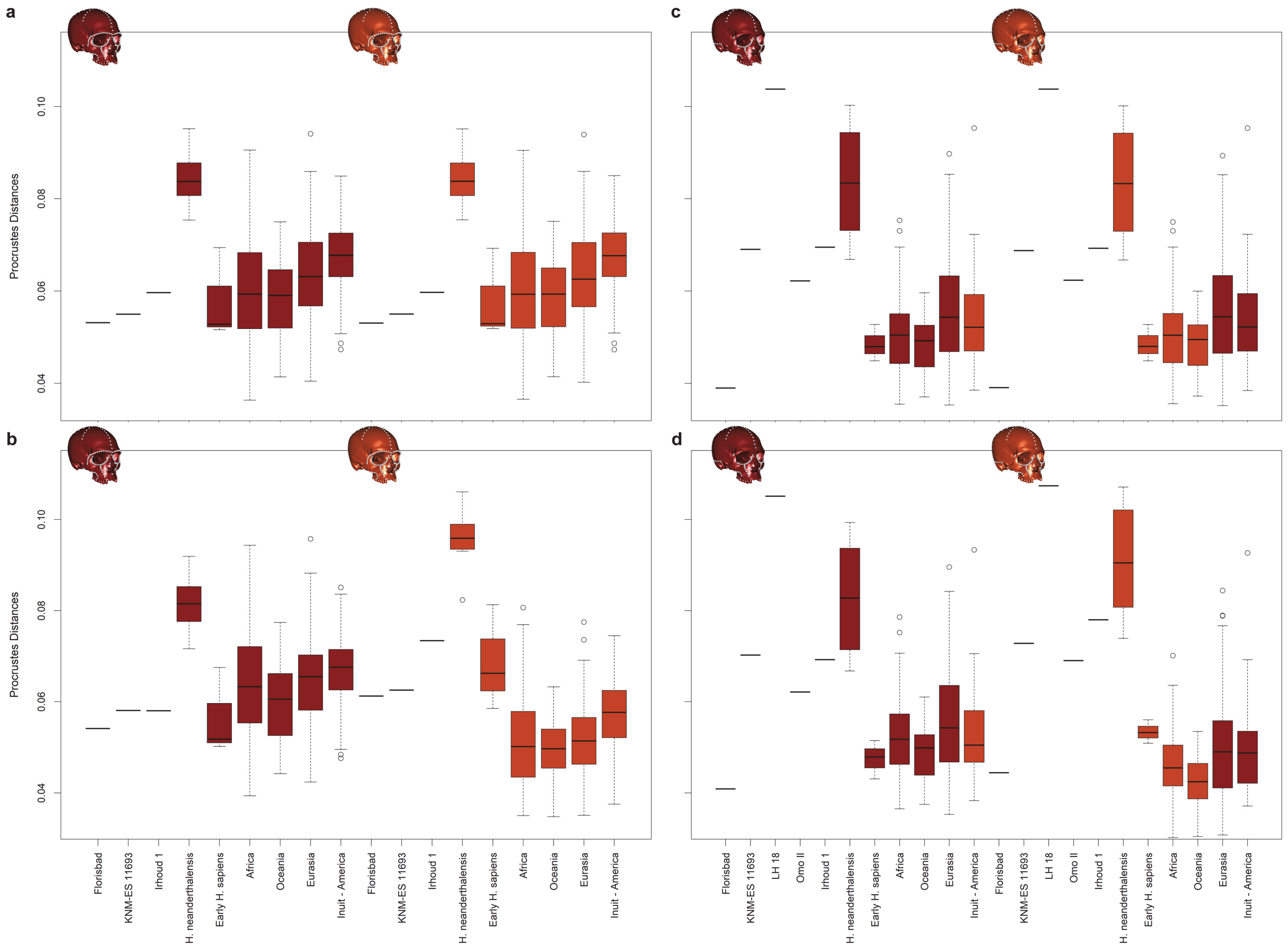

a
b
c
d
Procrustes Distances
0.04
0.06
0.08
0.10
Florisbad
KNM-ES 11693
LH 18
Omo II
Irhoud 1
H. neanderthalensis
Early H. sapiens
Africa
Oceania
Eurasia
Inuit - America

Supplementary Figure 5. Box-and-whisker plots showing the Procrustes distances between the vLCAs and the comparative sample for analyses C and D.

(a) and (c) show the distances to vLCAs 1 and 2. (b) and (d) present the distances to vLCAs 1b and 2b. Coloured boxes indicate the interquartile range (25 to 75%) around the median (vertical line); the dotted lines indicate the maximum and minimum range for each population, the circles are outliers.

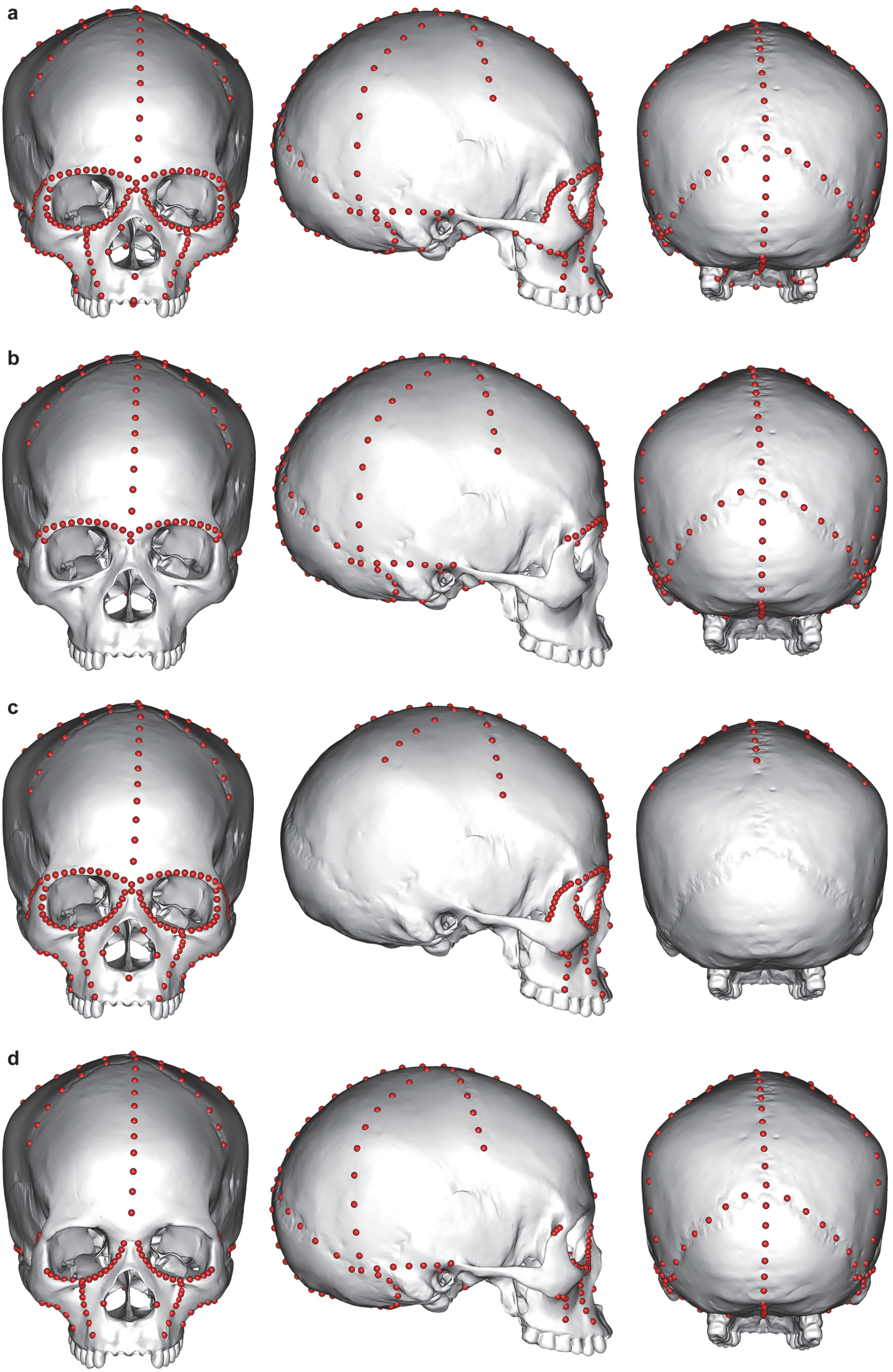

Supplementary Figure 6. Landmarks configurations used to run the GPAs and bgPCAs.

(a) analysis A: full skull, (b) analysis B: calvarium only, (c) analysis C: preserved data for Florisbad, and (d) analysis D: preserved data for KNM-ES 11693.

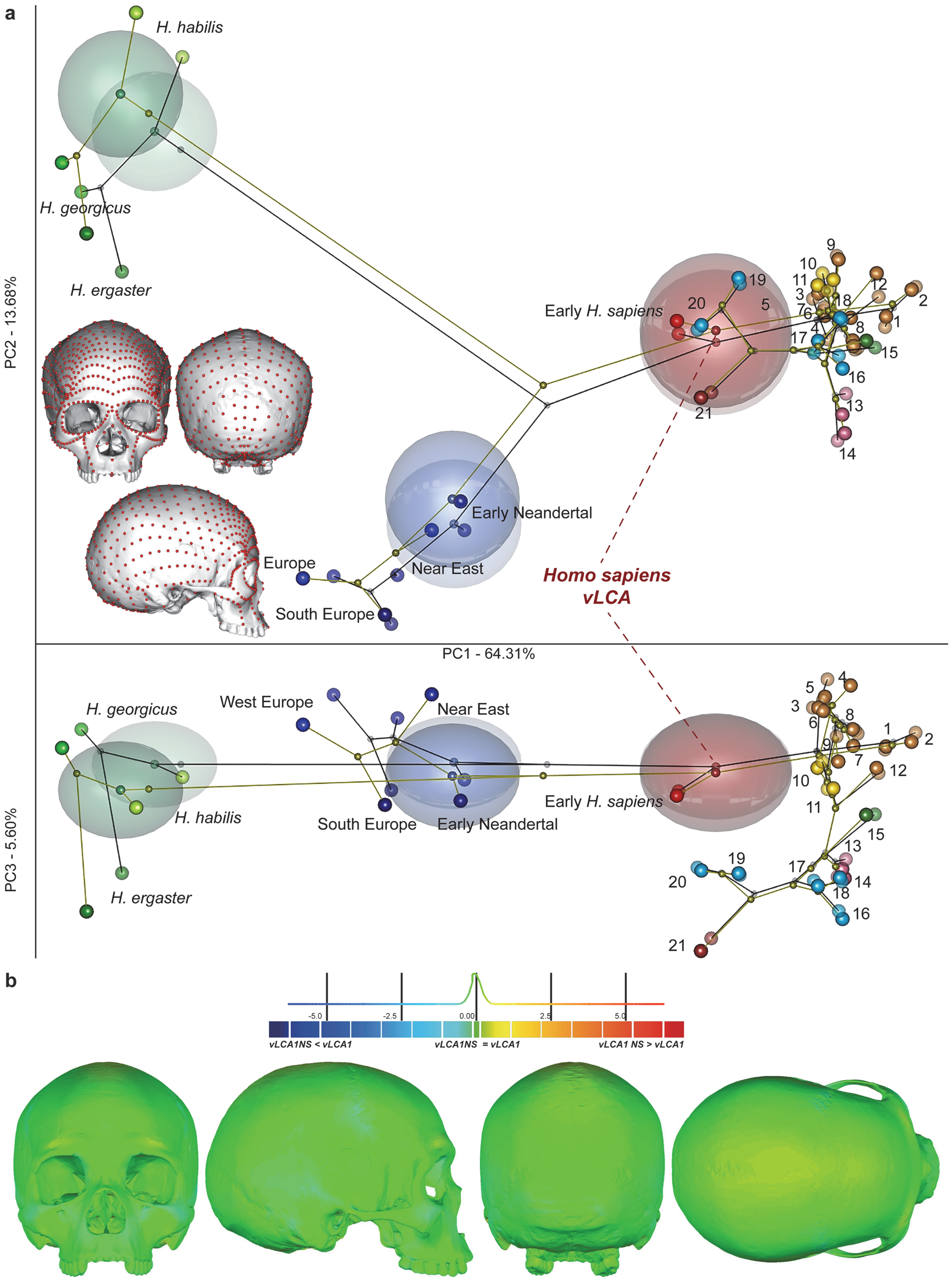

Supplementary Figure 7. Results from the modelling based on hypotheses 1 and 1NS.

(a) Projection of the phylogenies 1 (black tree) and 1NS based on non-slid semilandmarks (yellow tree) in the morphospace. Nodes represent the computed ancestors' shape and display the 95% confidence envelope. (b) Surface

deviation spectrum between vLCA1 and vLCA1NS. The difference in the shape between the vLCAs is < to 1.0 mm (see, Supplementary Table 6). Modern human populations as follow: 1 to 9 Sub-Saharan Africa; 10-11 Oceania, 12 North Africa, 13-14 Europe, 15 South Asia, 16 to 20 East Asia, 21 North America (see, Fig. 1 and Supplementary Data 1). Source data are provided as a Source Data file.

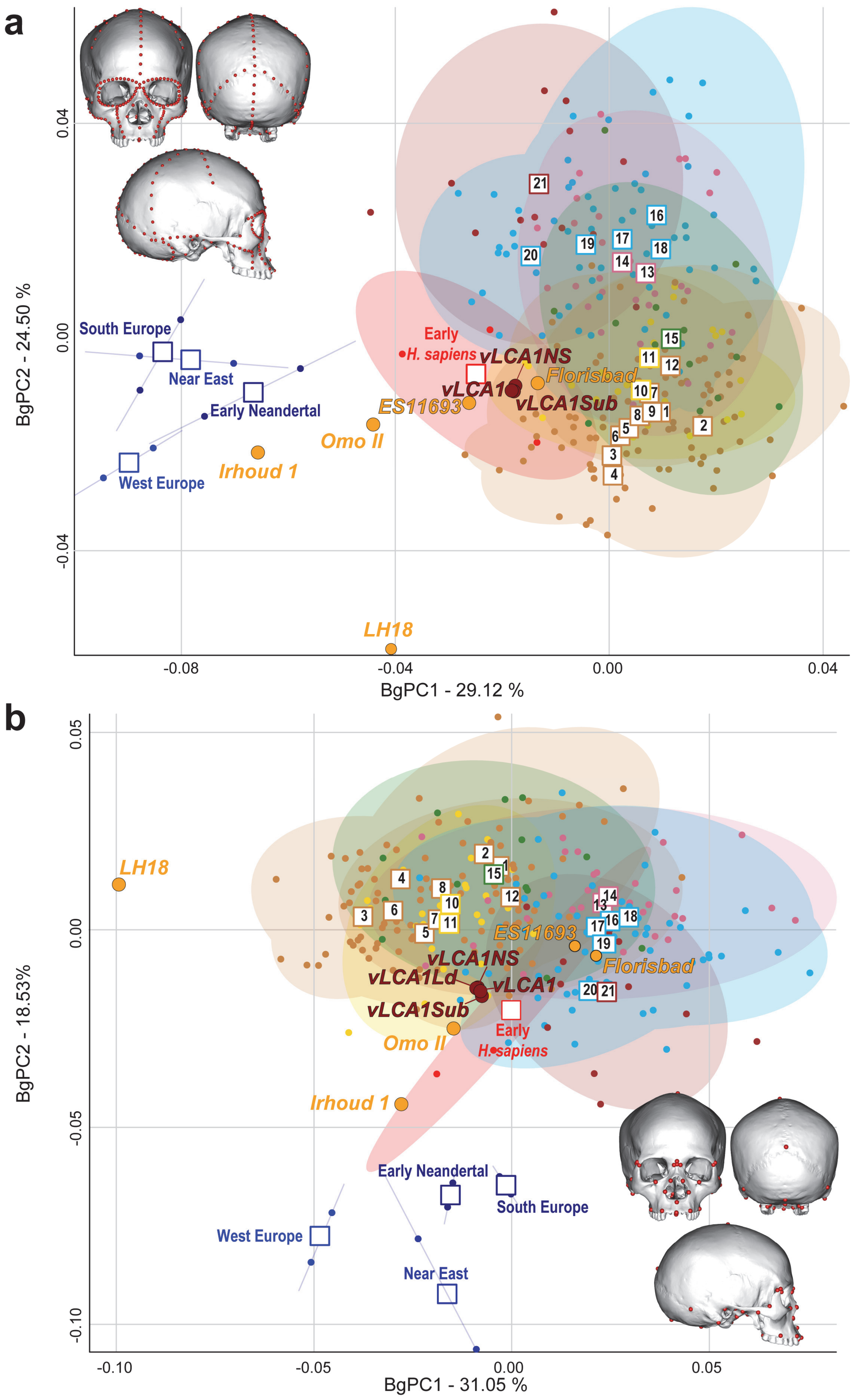
a
b
BgPC1 - 29.12 %
BgPC2 - 24.50 %
BgPC1 - 31.05 %
BgPC2 - 18.53%
South Europe
Near East
Early Neandertal
West Europe
Early
H. sapiens
vLCA1NS
vLCA1
vLCA1Sub
vLCA1Ld
Florisbad
ES11693
Omo II
Irhoud 1
LH18
-0.08
-0.04
0.00
0.04
-0.10
-0.05
0.05

Supplementary Figure 8. Morphospaces of bgPCAs for analyses E and F.

(a) shows the bgPCA from analysis E where the coordinates of the 239 landmarks and semilandmarks were extracted from vLCA1, vLCA1NS, vLCA1Sub as well as from the original comparative data from analysis A. (b) shows the bgPCA from analysis F which was run on 53 landmarks extracted from vLCA1, vLCA1NS, vLCA1Sub and vLCA1Ld along from the original comparative data from analysis A. The ellipses represent the 90% confidence interval for the estimated distribution of the specimens of each population. Both bgPCAs show that the differences between the ancestral shapes are small. Modern human populations as follow: 1 to 9 Sub-Saharan Africa; 10-11 Oceania, 12 North Africa, 13-14 Europe, 15 South Asia, 16 to 20 East Asia, 21 North America (see, Fig. 1 and Supplementary Data 1). Source data are provided as a Source Data file.

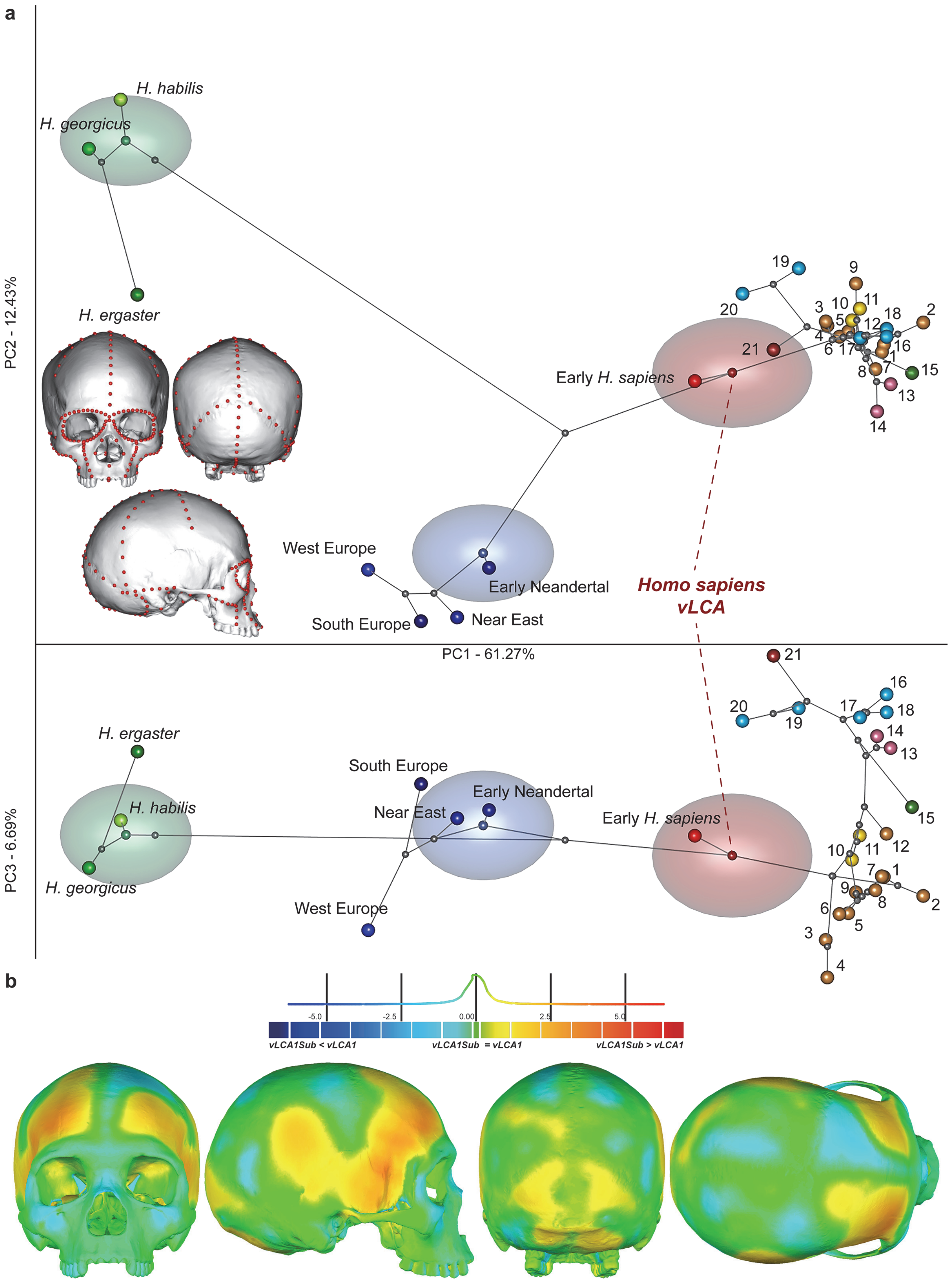

Supplementary Figure 9. Results from the modelling based on hypothesis 1Sub.

(a) Projection of the phylogeny 1Sub based on a sub-sample of 239 landmarks and semilandmarks in the morphospace. Nodes represent the computed ancestors' shape and display the 95% confidence envelope. (b) Surface deviation

spectrum between vLCA1 and vLCA1Sub. The difference in the shape between the vLCAs is < to 3.0 mm (see, Supplementary Table 6) for the areas where no landmark was inputted on the specimens used to compute vLCA1 Sub. Where landmarks were inputted, the difference in shape is < 1.0 mm. Modern human populations as follow: 1 to 9 Sub-Saharan Africa; 10-11 Oceania, 12 North Africa, 13-14 Europe, 15 South Asia, 16 to 20 East Asia, 21 North America (see, Fig. 1 and Supplementary Data 1). Source data are provided as a Source Data file.

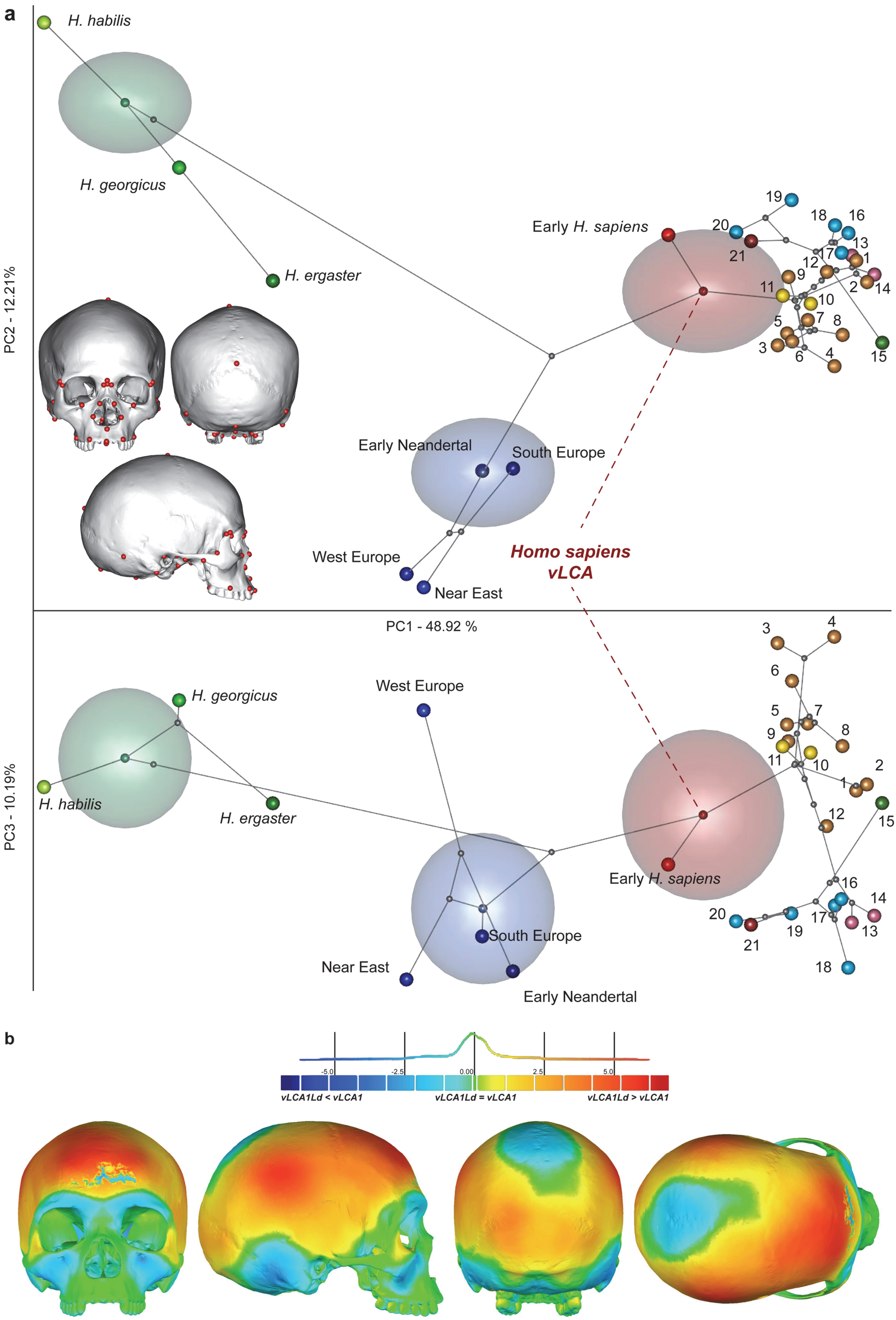
a
H. habilis
H. georgicus
H. ergaster
Early H. sapiens
Early Neandertal
South Europe
West Europe
Near East
Homo sapiens
vLCA
PC2 - 12.21%
PC1 - 48.92 %
PC3 - 10.19%
b
-5.0
-2.5
0.00
2.5
5.0
vLCA1Ld < vLCA1
vLCA1Ld = vLCA1
vLCA1Ld > vLCA1

Supplementary Figure 10. Results from the modelling based on hypothesis 1Ld.

(a) Projection of the phylogeny 1Ld based on a sub-sample of 53 landmarks in the morphospace. Nodes represent the computed ancestors' shape and display the 95% confidence envelope. (b) Surface deviation spectrum between vLCA1 and vLCA1Ld. The difference in the shape between the vLCAs is < to 6.0 mm (see, Supplementary Table 6) for the areas where no landmark was inputted on the specimens used to compute vLCA1 Ld. Where landmarks were inputted, the difference in shape is < 1.0 mm. Modern human populations as follow: 1 to 9 Sub-Saharan Africa; 10-11 Oceania, 12 North Africa, 13-14 Europe, 15 South Asia, 16 to 20 East Asia, 21 North America (see, Fig. 1 and Supplementary Data 1). Source data are provided as a Source Data file.

Supplementary References

93 Feibel, C. S., Brown, F. H. & McDougall, I. Stratigraphic context of fossil hominids from the Omo group deposits: Northern Turkana Basin, Kenya and Ethiopia. *Am. J. Phys. Anthropol.* **78**, 595-622, doi:10.1002/ajpa.1330780412 (1989).

94 McDougall, I. & Brown, F. H. Precise $^{40}Ar/^{39}Ar$ geochronology for the upper Koobi Fora Formation, Turkana Basin, northern Kenya. *J. Geol. Soc* **163**, 205-220, doi:https://doi.org/10.1144/0016-764904-166 (2006).

95 Gabunia, L., Vekua, A. & Lordkipanidze, D. The environmental contexts of early human occupation of Georgia (Transcaucasia). *J. Hum. Evol.* **38**, 785-802, doi:10.1006/jhev.1999.0383 (2000).

96 Marra, F. *et al.* A new age within MIS 7 for the *Homo neanderthalensis* of Saccopastore in the glacio-eustatically forced sedimentary successions of the Aniene River Valley, Rome. *Quat. Sci. Rev.* **129**, 260-274, doi:http://dx.doi.org/10.1016/j.quascirev.2015.10.027 (2015).

97 Vogel, J. C. & H.T., W. Radiocarbon. *Groningen Radiocarbon Dates IV* **5**, 163-202 (1963).

98 Oakley, K. P. The problem of man’s antiquity. An historical survey *Bulletin of the British Museum of Natural History. Geology Series* **9**, 85-155 (1964).

99 Blackwell, B. *et al.* ESR dating bovid teeth from the Neanderthal layer at La Ferrassie, France. *Geol. Soc. Am. Bull.* **39**, 548 (2007).

100 Hendrickx, S. La chronologie de la préhistoire tardive et des débuts de l'histoire de l'Egypte. *Archéo-Nil* **9**, 13-81 (1999).

101 Patin, E. *et al.* Dispersals and genetic adaptation of Bantu-speaking populations in Africa and North America. *Science* **356**, 543-546, doi:10.1126/science.aal1988 (2017).

102 Yang, M. A. & Fu, Q. Insights into Modern Human Prehistory Using Ancient Genomes. *Trends Genet.* **34**, 184-196, doi:https://doi.org/10.1016/j.tig.2017.11.008 (2018).

103 Nakagome, S. *et al.* Model-Based Verification of Hypotheses on the Origin of Modern Japanese Revisited by Bayesian Inference Based on Genome-Wide SNP Data. *Mol. Biol. Evol.* **32**, 1533-1543, doi:10.1093/molbev/msv045 (2015).

104 Raghavan, M. *et al.* Genomic evidence for the Pleistocene and recent population history of Native Americans. *Science* **349**, doi:10.1126/science.aab3884 (2015).

105 Blomberg, S. P., Garland, T. & Ives, A. R. TESTING FOR PHYLOGENETIC SIGNAL IN COMPARATIVE DATA: BEHAVIORAL TRAITS ARE MORE LABILE. *Evolution* **57**, 717-745, doi:10.1111/j.0014-3820.2003.tb00285.x (2003).

106 Copes, L. E. Comparative and Experimental Investigations of Cranial Robusticity in Mid-Pleistocene Hominins. Ph.D Dissertation thesis, Arizona State University, (2012).
