# Supplementary material for: Deciphering African late middle Pleistocene hominin diversity and the origin of our species
Source: Nat Commun. 2019 Sep 10;10:3406. doi: 10.1038/s41467-019-11213-w (PMC6736881; doi:10.1038/s41467-019-11213-w)
Supplement: Supplementary file 2 — Reporting Summary [file 41467_2019_11213_MOESM2_ESM.pdf]

nature research

Corresponding author(s): Aurélien Mounier
Last updated by author(s): 08/05/2019

Reporting Summary

Nature Research wishes to improve the reproducibility of the work that we publish. This form provides structure for consistency and transparency in reporting. For further information on Nature Research policies, see Authors & Referees and the Editorial Policy Checklist .

Statistics

For all statistical analyses, confirm that the following items are present in the figure legend, table legend, main text, or Methods section.

n/a	Confirmed	
☐	☒	The exact sample size (*n*) for each experimental group/condition, given as a discrete number and unit of measurement
☐	☒	A statement on whether measurements were taken from distinct samples or whether the same sample was measured repeatedly
☒	☐	The statistical test(s) used AND whether they are one- or two-sided<br>*Only common tests should be described solely by name; describe more complex techniques in the Methods section.*
☐	☒	A description of all covariates tested
☒	☐	A description of any assumptions or corrections, such as tests of normality and adjustment for multiple comparisons
☐	☒	A full description of the statistical parameters including central tendency (e.g. means) or other basic estimates (e.g. regression coefficient) AND variation (e.g. standard deviation) or associated estimates of uncertainty (e.g. confidence intervals)
☒	☐	For null hypothesis testing, the test statistic (e.g. *F*, *t*, *r*) with confidence intervals, effect sizes, degrees of freedom and *P* value noted<br>*Give P values as exact values whenever suitable.*
☒	☐	For Bayesian analysis, information on the choice of priors and Markov chain Monte Carlo settings
☐	☒	For hierarchical and complex designs, identification of the appropriate level for tests and full reporting of outcomes
☒	☐	Estimates of effect sizes (e.g. Cohen's *d*, Pearson's *r*), indicating how they were calculated

Our web collection on statistics for biologists contains articles on many of the points above.

Software and code

Policy information about availability of computer code

Data collection	We used the Landmark Software (IDAV) to collect the 3D coordinates from the 3D models
Data analysis	We used Geomagic Studio v.2013.0.1 to perform the surface deviation spectrum analysis and R for all other analyses (Morpho v2.1; geomorph v2.1.2; ape v3.2; phytools v0.4-3.1, Ade4 v1.7-4, phylocurve v2.0.9).

For manuscripts utilizing custom algorithms or software that are central to the research but not yet described in published literature, software must be made available to editors/reviewers. We strongly encourage code deposition in a community repository (e.g. GitHub). See the Nature Research guidelines for submitting code & software for further information.

Data

Policy information about availability of data

All manuscripts must include a data availability statement. This statement should provide the following information, where applicable:

- Accession codes, unique identifiers, or web links for publicly available datasets
- A list of figures that have associated raw data
- A description of any restrictions on data availability

The 3D model of the vLCAs are available in Supplementary Data 5-11 along with the 3D models showing the deviation patterns between the vLCAs (Supplementary Data 12-18). The 3D geometric morphometric data (i.e. landmarks coordinates) are available in Source Data File.

Field-specific reporting

Please select the one below that is the best fit for your research. If you are not sure, read the appropriate sections before making your selection.

Ecological, evolutionary & environmental sciences study design

All studies must disclose on these points even when the disclosure is negative.

Study description	This study uses a phylogenetic modelling method to predict possible morphologies of a last common ancestor of all modern humans, which are used to understand human evolution at the end of the Middle Pleistocene. The possible ancestors are compared to the Late Middle Pleistocene African fossils (KNM-ES 11693, Florisbad, Irhoud 1, Omo II, and LH18), using 3D geometric morphometrics. Our results support a complex process for the evolution of Homo sapiens, with the recognition of different populations and lineages in Africa – not all of which contributed to our species' origin.
Research sample	The sample is made of 6 individuals of early Homo, 8 Homo neanderthalensis, 249 Homo sapiens and 5 Late Middle Pleistocene hominin crania. The specimens are described with landmarks and semilandmarks.
Sampling strategy	Regarding the fossil hominin specimens, we tried to obtain access to the majority of most well-preserved fossil specimens. Regarding the modern human sample we aimed at having sex-balanced groups of 10-15 individuals for each population included in the study (n=21).
Data collection	3D coordinates (landmarks and semilandmarks) were collected by AM using the Landmark Software (IDAV) from 3D models which were obtained by AM from photogrammetry, CT-scan segmentation or opticla scanning.
Timing and spatial scale	The landmarks and semi-landmarks were collected in March and April 2017 for 58 of the specimens. The other specimens were collected in February and March 2018 following comments from reviewers of the paper.
Data exclusions	No data were excluded from the analysis.
Reproducibility	Three additional models have been run on subsample and/or different landmarks 3D coordinates (non-slid) to ensure the strength of the results (see supplementary information).
Randomization	The specimens are grouped according to their populations of origin.
Blinding	There was no blinding during data acquistion. Data were collected by AM (3D models building and 3D coordinates).

Did the study involve field work? ☐ Yes ☒ No

Reporting for specific materials, systems and methods

We require information from authors about some types of materials, experimental systems and methods used in many studies. Here, indicate whether each material, system or method listed is relevant to your study. If you are not sure if a list item applies to your research, read the appropriate section before selecting a response.

Materials & experimental systems

n/a	Involved in the study
☒	☐ Antibodies
☒	☐ Eukaryotic cell lines
☒	☐ Palaeontology
☒	☐ Animals and other organisms
☒	☐ Human research participants
☒	☐ Clinical data

Methods

n/a	Involved in the study
☒	☐ ChIP-seq
☒	☐ Flow cytometry
☒	☐ MRI-based neuroimaging
