# Supplementary material for: Deciphering African late middle Pleistocene hominin diversity and the origin of our species
Source: Nat Commun. 2019 Sep 10;10:3406. doi: 10.1038/s41467-019-11213-w (PMC6736881; doi:10.1038/s41467-019-11213-w)
Supplement: Supplementary file 3 — Description of Additional Supplementary Files [file 41467_2019_11213_MOESM3_ESM.pdf]

Description of Additional Supplementary Files

Supplementary Data 1. Euclidean distances between group means of bgPCA analysis A

Supplementary Data 2. Euclidean distances between group means of bgPCA analysis B

Supplementary Data 3. Euclidean distances between group means of bgPCA analysis C

Supplementary Data 4. Euclidean distances between group means of bgPCA analysis D

Supplementary Data 5. 3d model of vLCA1 (.ply) *

Supplementary Data 6. 3d model of vLCA2 (.ply) *

Supplementary Data 7. 3d model of vLCA1b (.ply) *

Supplementary Data 8. 3d model of vLCA2b (.ply) *

Supplementary Data 9. 3d model of vLCA1NS (.ply) *

Supplementary Data 10. 3d model of vLCA1Sub (.ply) *

Supplementary Data 11. 3d model of vLCA1Ld (.ply) *

Supplementary Data 12. Surface deviation of vLCA1 vs 2 (.ply, see Fig. 3a) *

Supplementary Data 13. Surface deviation of vLCA1 vs 1b (.ply, see Supplementary Fig. 3a) *

Supplementary Data 14. Surface deviation of vLCA2 vs 2b (.ply, see Supplementary Fig. 3b) *

Supplementary Data 15. Surface deviation of vLCA1b vs 2b (.ply, see Supplementary Fig. 3c) *

Supplementary Data 16. Surface deviation of vLCA1 vs 1NS (.ply, see Supplementary Fig. 7) *

Supplementary Data 17. Surface deviation of vLCA1 vs 1Sub (.ply, see Supplementary Fig. 8) *

Supplementary Data 18. Surface deviation of vLCA1 vs 1Ld (.ply, see Supplementary Fig. 9) *

*In order to visualise properly Supplementary Data 5-18, please use the open source Meshlab software [http://www.meshlab.net/#download] or use the R software with the following command lines where X stands for the drive letter and Y stands for the number of the Supplementary Data file (i.e. 5 to 18):

```
# open Libraries
library(rgl)
library(Morpho)
# import Mesh
Mesh <- file2mesh('X:/SuppDataY.ply', readcol=TRUE)
# view imported Mesh
open3d()
shade3d(Mesh)
```
